# The expression of mouse CLEC‐2 on leucocyte subsets varies according to their anatomical location and inflammatory state

**DOI:** 10.1002/eji.201445314

**Published:** 2015-08-12

**Authors:** Kate L. Lowe, Leyre Navarro‐Núñez, Cécile Bénézech, Saba Nayar, Bethany L. Kingston, Bernhard Nieswandt, Francesca Barone, Steve P. Watson, Christopher D. Buckley, Guillaume E. Desanti

**Affiliations:** ^1^ Centre for Cardiovascular SciencesUniversity of BirminghamBirminghamUK; ^2^ MRC Centre for Immune RegulationUniversity of BirminghamBirminghamUK; ^3^ Centre for Translational Inflammation ResearchRheumatology Research GroupUniversity of BirminghamBirminghamUK; ^4^ Medical SchoolUniversity of OxfordOxfordUK; ^5^ Department of Experimental BiomedicineUniversity Hospital, University of WürzburgWürzburgGermany; ^6^ BHF Centre for Cardiovascular ScienceUniversity of EdinburghEdinburghUK

**Keywords:** CLEC‐2, Inflammation, Leucocytes, Mouse, Tamoxifen

## Abstract

Expression of mouse C‐type lectin‐like receptor 2 (CLEC‐2) has been reported on circulating CD11b^high^ Gr‐1^high^ myeloid cells and dendritic cells (DCs) under basal conditions, as well as on a variety of leucocyte subsets following inflammatory stimuli or in vitro cell culture. However, previous studies assessing CLEC‐2 expression failed to use CLEC‐2‐deficient mice as negative controls and instead relied heavily on single antibody clones. Here, we generated CLEC‐2‐deficient adult mice using two independent approaches and employed two anti‐mouse CLEC‐2 antibody clones to investigate surface expression on hematopoietic cells from peripheral blood and secondary lymphoid organs. We rule out constitutive CLEC‐2 expression on resting DCs and show that CLEC‐2 is upregulated in response to LPS‐induced systemic inflammation in a small subset of activated DCs isolated from the mesenteric lymph nodes but not the spleen. Moreover, we demonstrate for the first time that peripheral blood B lymphocytes present exogenously derived CLEC‐2 and suggest that both circulating B lymphocytes and CD11b^high^ Gr‐1^high^ myeloid cells lose CLEC‐2 following entry into secondary lymphoid organs. These results have significant implications for our understanding of CLEC‐2 physiological functions

## Introduction

Many physiological functions are critically regulated by fine‐tuned interactions between diverse subsets of hematopoietic and nonhematopoietic cells within primary and secondary lymphoid organs (SLOs) as well as in the circulation. These interactions are mediated by surface receptors or secreted molecules that display complex cellular, spatial, and temporal expression patterns. A deep understanding of these patterns is required for a full knowledge of their physiological significance and for effective therapeutic intervention. For example, expression of receptor activator of NFκB ligand by bone osteoblasts regulates bone density while its expression by developing thymocytes regulates medullary thymic epithelial cell maturation (for review; 1).

A similar scenario can be considered for the C‐type lectin‐like receptor 2 (CLEC‐2) and its ligand podoplanin (PDPN), which are involved in a variety of physiological and pathophysiological processes (for review: 2). While CLEC‐2 expression is restricted to hematopoietic cells, PDPN is more ubiquitously expressed, with constitutive expression in the lungs 3, kidneys 4, brain 3, thymus 5, SLOs 6, 7, lymphatic vessels, and bones 8. PDPN expression is also upregulated at the leading edge of tumors and in additional hematopoietic and nonhematopoietic cell types during inflammation (for review: 2). CLEC‐2/PDPN interactions play essential roles in the immune system, as they prevent blood–lymph mixing 9, 10, 11, are required for lymph node (LN) development 12 and maintenance of LN vascular integrity 12, 13 and contribute to the generation of optimal adaptive immune responses 12, 14, 15.

CLEC‐2 surface expression on platelets was first demonstrated in humans 16 and soon after in mouse 17 and chicken 18. The expression of CLEC‐2 and its RNA transcript – encoded by the C‐type lectin domain family 1, member B *(Clec1b)* gene – has also been studied in leucocytes isolated from different species leading to a rather confusing mosaic of results. While CLEC‐2 is absent from chicken leucocytes 18 and restricted to liver‐resident Küppfer cells in human 19, 20, 21, 22, a much broader expression profile of CLEC‐2/*Clec1b* has been reported in rodent leucocytes, particularly in mice.

While one report claims that mouse CLEC‐2 surface expression by leucocytes is restricted to monocytes and liver‐resident Küppfer cells 20, other studies using a different antibody clone (17D9), or the fusion protein PDPN‐Fc, reported that CLEC‐2 is constitutively expressed by CD11b^high^ Gr‐1^high^ cells isolated from bone marrow (BM) and whole blood, splenic B lymphocytes, a small subset of splenic natural killer (NK) cells, splenic plasmacytoid dendritic cells (pDCs), splenic conventional DCs (cDCs), GM‐CSF stimulated BM‐derived DCs (BMDCs), Flt3L BMDCs, as well as peripheral LN DCs 19, 23, 24. With the exception of NKT cells and T lymphocytes, in vivo LPS challenge has been reported to upregulate CLEC‐2 expression in almost all splenic leucocyte subsets as well as peripheral LN DCs 23, 24. In a thioglycolate‐induced peritoneal inflammation model, CLEC‐2 expression was observed in F4/80^+^ macrophages but not in CD11b^high^ Gr‐1^high^ cells 19, 23. Notably, CLEC‐2‐deficient negative control cells were not included in most of these studies 19, 23. Our study aimed to clarify these contradictory findings and improve our understanding of CLEC‐2 expression on mouse leucocytes. These results have important physiological consequences that will be discussed below.

## Results and discussion

### Peripheral blood B lymphocytes and CD11b^high^ Gr‐1^high^ cells present CLEC‐2 on their surface

Previous studies that investigated the temporal, spatial, and proinflammatory expression of CLEC‐2 in the murine adult hematopoietic system have been hampered by the high neonatal mortality rate (>95%) of *Clec1b^−/−^* mice 10, 20, impeding the inclusion of appropriate *Clec1b^−/−^* negative control cells in previous studies aiming to define the temporal, spatial, and postinflammatory expression of CLEC‐2 in vivo 19, 23, 24.

To circumvent the neonatal mortality rate of *Clec1b^−/−^* mice, we developed a tamoxifen‐inducible *Clec1b* deleting mouse line (*Clec1b^fl/fl^xRosa26^+/creERT2^*). After 6 months on tamoxifen diet, peripheral blood leucocytes isolated from *Clec1b^fl/fl^xRosa26^+/creERT2^* mice but not *Clec1b^fl/fl^* littermate controls show genomic deletion of the *Clec1b* locus (Supporting Information Fig. 1).

In parallel, we investigated CLEC‐2 expression on hematopoietic cells isolated from lethally irradiated wild‐type (WT) adult mice reconstituted with foetal liver (FL) cells from E14.5 *Clec1b^+/+^* or *Clec1b^−/−^* embryos 25. This second experimental strategy was used to rule out potential side effects of tamoxifen on CLEC‐2 expression. It is known that sex steroid hormones and their synthetic derivatives (such as tamoxifen) affect hematopoiesis due to the presence of estrogen receptors on most immune cells 26, 27. Moreover, tamoxifen has anti‐inflammatory effects that could counteract LPS‐mediated proinflammatory challenges 28, 29, 30. In addition, we used two different antibody clones, 17D9 19, 23 and INU1 31, reported to bind to mouse CLEC‐2.

**Figure 1:**
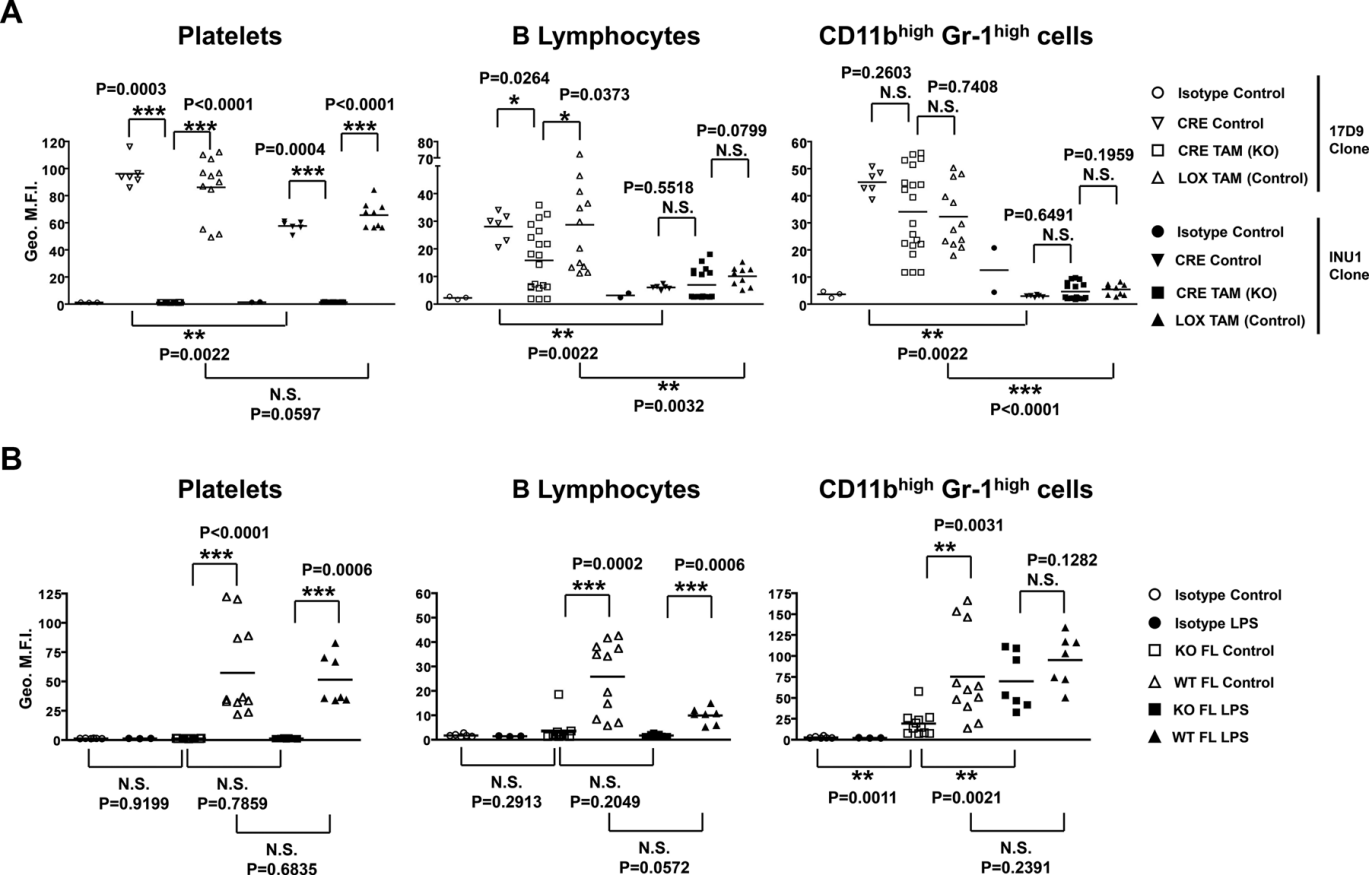
CLEC‐2 is present at the surfaces of peripheral blood platelets, B cells, and CD11b^high^ Gr‐1^high^ cells at steady state. (A) *Clec1b^fl/fl^*x*Rosa26^+/creERT2^* (*CRE TAM*) and *Clec1b^fl/fl^* littermate controls (*LOX TAM*) were fed tamoxifen‐supplemented diet from 6–8 weeks old for 6 months. *Clec1b^fl/fl^*x*Rosa26^+/creERT2^* mice (11–14 weeks old) fed conventional diet only were used as controls (*CRE Control*). Blood was drawn from the tail vein, the erythrocytes were lysed and the remaining leucocytes stained with fluorochrome‐conjugated antibodies. CLEC‐2 expression on platelets, B lymphocytes, and myeloid cells was assessed using the 17D9 (white symbols) or INU1 (black symbols) antibody clones compared to their respective isotype controls by flow cytometry. The staining intensities are expressed by the geometric mean of fluorescence intensity (*Geo. M.F.I*). (B) Wild‐type (WT) animals were lethally irradiated (2 × 450 rad) and injected i.v. with FL cells from *Clec1b^+/+^* (*WT FL*) or *Clec1b^−/−^* (*KO FL*) E14.5 embryos. Six to eight weeks postinjection animals were challenged with 25 μg LPS by i.p. injection (*WT FL LPS* or *KO FL LPS*, *black symbols*) and compared to nonchallenged animals (*WT FL Control* or *KO FL Control*, *white symbols*). Sixteen to eighteen hours post LPS injection, blood was harvested by full exsanguination and processed as described above. Each symbol represents a sample from an individual mouse. Bars represent the means. The graphs summarize one to three independent experiments pooled together. Statistical significance was measured by a Mann–Whitney test with a 95% confidence interval, where: *p < 0.05; **p < 0.005; ***p < 0.0005; N.S., not significant.

Initially, CLEC‐2 expression was measured on circulating platelets, T lymphocytes, B lymphocytes, and CD11b^high^ Gr‐1^high^ cells from *Clec1b^fl/fl^xRosa26^+/creERT2^* mice and *Clec1b^fl/fl^* littermates by flow cytometry using the two antibody clones 17D9 and INU1 (Fig. 1A and Supporting Information Fig. 2). Following tamoxifen treatment, *Clec1b^fl/fl^xRosa26^+/creERT2^* platelets showed full abrogation of CLEC‐2 expression compared to *Clec1b^fl/fl^* littermates using both 17D9 and INU1 (Fig. 1A), confirming the efficiency of our inducible genetic mouse model.

On platelets, INU1 staining was found to be weaker than 17D9 staining in both control animals (*Clec1b^fl/fl^* mice treated with tamoxifen and *Clec1b^fl/fl^xRosa26^+/creERT2^* mice fed with normal diet). Furthermore, the geometric mean fluorescence intensity associated with 17D9 binding to leucocytes was on average three‐fold lower than that observed on platelets (Fig. 1A), while INU1 discrimination power was too weak for detecting CLEC‐2 on leucocytes (Fig. 1A). As a result, we solely used the 17D9 clone to further investigate CLEC‐2 expression on leucocytes.

In both the tamoxifen‐inducible and radiation chimeric CLEC‐2‐deficient mouse models, the levels of 17D9 binding to circulating B lymphocytes were significantly reduced compared to controls (Fig. 1A and B), suggesting that peripheral blood B lymphocytes constitutively express CLEC‐2. Although CLEC‐2 appeared to be downregulated on circulating B lymphocytes following LPS treatment in chimeric mice, this was not statistically significant (Fig. 1B), indicating that activated *Clec1b^+/+^* B lymphocytes remain positive for CLEC‐2 when compared to their activated *Clec1b^−/−^* B lymphocyte counterparts.

In the absence of LPS‐induced inflammation, CD11b^high^ Gr‐1^high^ cells (which includes a mix of monocytes, granulocytes/neutrophils, and a small subset of NK cells 32, 33) isolated from *Clec1b^−/−^* reconstituted animals were negative for CLEC‐2 compared to *Clec1b^+/+^* littermates (Fig. 1B). This demonstrates that circulating CD11b^high^ Gr‐1^high^ cells constitutively express CLEC‐2 in line with previous reports 19, 23. However, treatment with tamoxifen led to the loss of specific CLEC‐2 staining, since no significant difference was observed between *Clec1b^fl/fl^xRosa26^+/creERT2^* mice and their controls (Fig. 1A). Tamoxifen is known to inhibit B lymphocyte and DC maturation by altering the surface membrane expression of molecules such as CD22 on B lymphocytes or MHC‐II and CD86 on BMDCs 34, 35. Additional uncharacterized phenotypic changes leading to the appearance of new CLEC‐2‐independent binding sites might explain the nonspecific binding of 17D9 to *Clec1b^fl/fl^xRosa26^+/creERT2^* B lymphocytes and CD11b^high^ Gr‐1^high^ cells as the WT *Clec1b* DNA was undetectable in these cells (Fig. 1A, Supporting Information Fig. 1).

Similarly, after LPS challenge, 17D9 acquired the ability to bind to *Clec1b^−/−^* CD11b^high^ Gr‐1^high^ cells leading to a 3.7‐fold higher geometric mean fluorescence intensity than in equivalent unstimulated cells (Fig. 1B). This suggests that 17D9 binds to CD11b^high^ Gr‐1^high^ cells in a CLEC‐2‐independent manner following LPS activation. In T lymphocytes, there was no evidence for 17D9 binding in any of our experimental conditions (Supporting Information Fig. 3).

From these findings, we suggest that mouse peripheral blood B lymphocytes and CD11b^high^ Gr‐1^high^ cells present CLEC‐2 on their surface at a much lower level than platelets. These data contrast with the lack of evidence for CLEC‐2 expression in peripheral blood leucocytes in chickens or humans 18, 21 and with the absence of significant *Clec1b* transcripts in human leucocyte subsets according to microarray analyses on the BioGPS database 21, 36. This indicates that, while CLEC‐2 expression by platelets is conserved through species, the presence of CLEC‐2 on B lymphocytes and CD11b^high^ Gr‐1^high^ cells is specific to mice. Whether the presence of CLEC‐2 on these cells provides any particular features to the mouse immunological system remains unknown. Our data add to previous reports establishing important differences between the mouse and human immune systems 37.

### Most SLO‐resident leucocytes do not express CLEC‐2/Clec1b at steady state

**Figure 2:**
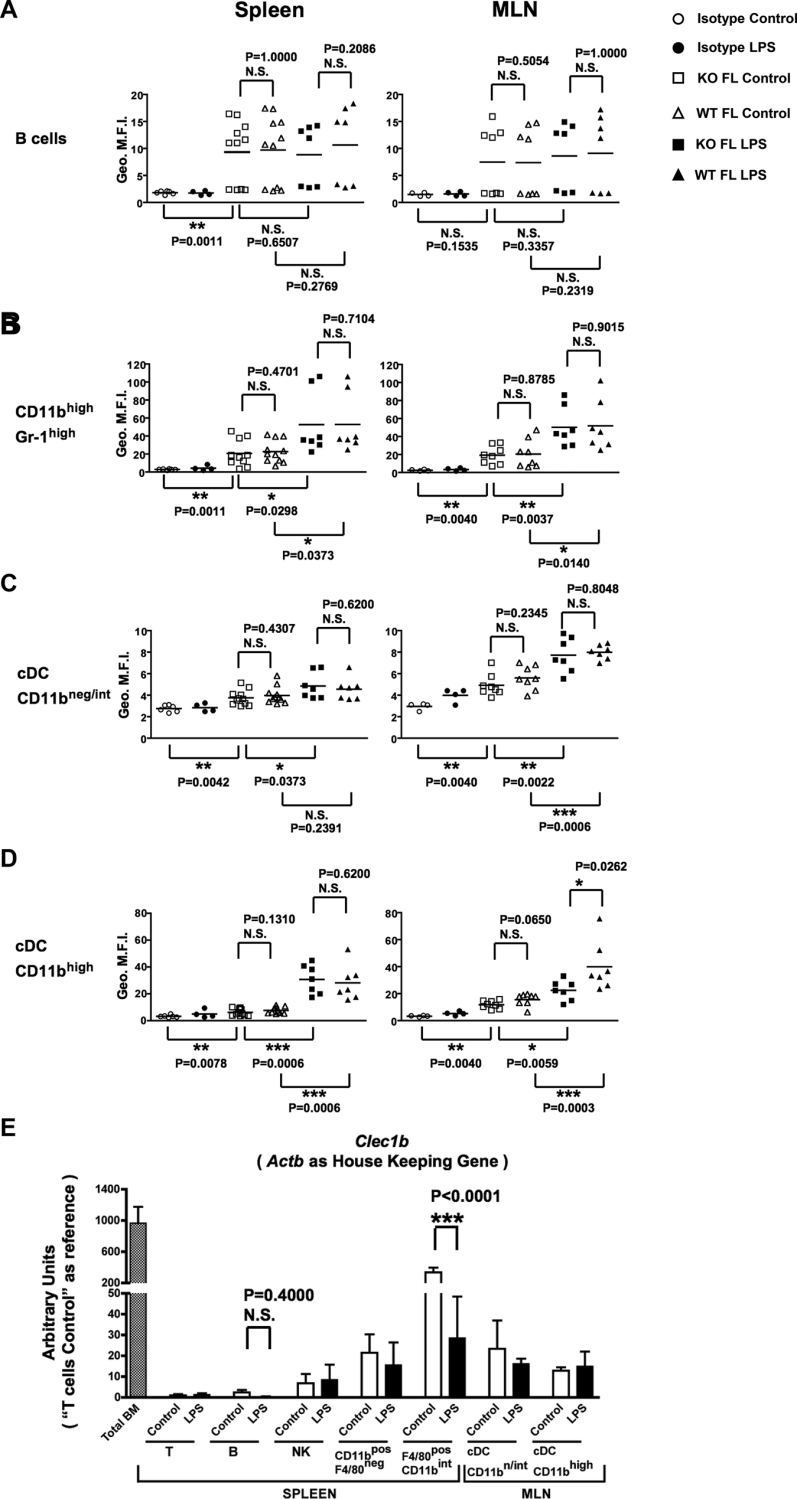
In SLOs, CLEC‐2 expression by leucocytes is restricted to a subpopulation of activated DCs. At 6–8 weeks old, *Clec1b^+/+^* (*WT FL*) or *Clec1b^−/−^* (*KO FL*) FL chimeras were challenged with 25 μg LPS by i.p. injection (*WT FL LPS* or *KO FL LPS*, *black symbols*) and compared to nonchallenged animals (*WT FL Control* or *KO FL Control*, *white symbols*). Sixteen to eighteen hours after LPS injections, the spleen and MLN were harvested, the erythrocytes were lysed and the remaining leucocytes stained with fluorochrome‐conjugated antibodies. CLEC‐2 expression on (A) B cells, (B) CD11b^high^ Gr‐1^high^ cells, (C) CD11b^neg/int^ DCs, and (D) CD11b^high^ DCs was assessed using the 17D9 antibody compared to its respective isotype control by flow cytometry. The staining intensities are expressed as the geometric mean of fluorescence intensity (*Geo. M.F.I*). Each symbol represents a sample from an individual mouse and bars represent means. The graphs summarize three independent experiments pooled together. (E) Relative expression of *Clec1b* transcript in leucocytes isolated from the spleen and the MLN. C57BL/6 mice were injected i.p. with PBS (*white bars*) or 25 μg LPS (*black bars*). Sixteen and eighteen hours later the spleen and MLN were harvested, the erythrocytes were lysed and the remaining leucocytes stained. Leucocyte populations were isolated by FACS based on the following phenotypes: T cells (T): DAPI^neg^ CD41^neg^ F4/80^neg^ CD11c^neg^ CD19^neg^ B220^neg^ CD3ε^pos^ CD8α/CD4^pos^; B cells (B): DAPI^neg^ CD41^neg^ F4/80^neg^ CD11c^neg^ CD8α^neg^ CD3ε^neg^ B220^pos^ CD19^pos^; NK cells (NK): DAPI^neg^ CD41^neg^ CD19^neg^ CD8α^neg^ CD4^neg^ CD3ε^neg^ NK1.1/NKp46^pos^; CD11b^pos^ F4/80^neg^ cells: DAPI^neg^ CD41^neg^ CD19^neg^ CD3ε^neg^ F4/80^neg^ CD11b^pos^; F4/80^pos^ CD11b^int^ cells: DAPI^neg^ CD41^neg^ CD19^neg^ CD3ε^neg^ CD11b^int^ F4/80^pos^; cDC CD11b^neg/int^ cells: DAPI^neg^ CD41^neg^ CD19^neg^ CD3ε^neg^ B220^neg^ CD11c^pos^ CD11b^neg/int^; cDC CD11b^high^ cells: DAPI^neg^ CD41^neg^ CD19^neg^ CD3ε^neg^ B220^neg^ CD11c^pos^ CD11b^high^. After mRNA isolation and cDNA preamplification for the genes of interest, the samples were analyzed by quantitative PCR. The signal for *Clec1b* was normalized against the house‐keeping gene *Actnb* (β‐actin). *Total BM* isolated from PBS‐injected mice was used as positive control while the T cells coming from these animals were used as reference to set the arbitrary unit. Each population was isolated from three to five independent cell sorting experiments including one LPS‐injected and one PBS‐injected mouse for each cell sorting, with the exception of the F4/80^pos^ CD11b^int^ control and LPS‐stimulated cells for which *n* = 2 experiments. Data are shown as mean + SEM. Statistical significance was measured by a Mann–Whitney test with a 95% confidence interval where: *p < 0.05; **p < 0.005; ***p < 0.0005; N.S., not significant.

At steady state, we observed comparable 17D9 binding to spleen and mesenteric LN (MLN) B lymphocytes, CD11b^high^ Gr‐1^high^ cells, pDCs, CD11b^neg/int^ cDCs, and CD11b^high^ cDCs isolated from *Clec1b^−/−^* radiation chimeras or their WT counterparts (Fig. 2, Supporting Information Figs. 4 and 5). These results, that demonstrate a lack of CLEC‐2 expression on these leucocytes, contradict previous observations made using the 17D9 antibody clone and PDPN‐Fc recombinant protein that suggested that mouse CLEC‐2 was constitutively expressed on all these cell types in spleen and on peripheral LNs’ cDCs 23, 24. Moreover, we could not detect significant *Clec1b* transcript levels in most of the leucocyte populations isolated from the spleen and the MLN (Fig. 2E). The absence of *Clec1b* transcripts in most resting leucocytes is supported by two independent microarray analyses performed by the ImmGen 38 and BioGPS 36 consortia (Supporting Information Fig. 6).

Interestingly, in agreement with the ImmGen database, we did observe that CD11b^int^ F4/80^pos^ red pulp splenic macrophages express high levels of *Clec1b* transcripts (Fig. 2E). However, we were unable to detect surface CLEC‐2 on the surface of these cells when comparing our FL‐reconstituted animals (data not shown). Given that F4/80^pos^ red pulp splenic macrophages, which express PDPN, play a key physiological role in the clearance of senescent blood erythrocytes and platelets by phagocytosis 6, 39, 40, the *Clec1b* transcripts detected in these cells are likely to derive from engulfed platelets.

These results suggest that CLEC‐2 surface expression by peripheral blood B lymphocytes and CD11b^high^ Gr‐1^high^ cells (Fig. 1) is lost upon entry into SLOs (Fig. 2). The majority of mouse B lymphocytes and pre‐cDC monocytes migrating from peripheral blood toward LNs enter via high endothelial venules (HEVs), arriving in the T‐cell zone, which is rich in PDPN^pos^ fibroblastic reticular cells (FRCs) 41. Downregulation or shedding of CLEC‐2 by B lymphocytes and CD11b^high^ Gr‐1^high^ cells during their entry through the HEVs might represent a functional mechanism to prevent inappropriate activation of PDPN^pos^ FRCs in the absence of infection. In this context, recent studies demonstrated that close interactions between antigen‐activated CLEC‐2^pos^ DCs and LN PDPN^pos^ FRCs are required for mounting an effective immune response by favoring DC recruitment, FRCs activation and LN swelling 14, 15, 24.

### Most SLO‐resident leucocytes remain CLEC‐2‐negative following LPS stimulation

CLEC‐2 was not upregulated on splenic and MLN‐resident B lymphocytes, CD11b^high^ Gr‐1^high^ cells, pDCs, or CD11b^neg/int^ cDCs in response to intraperitoneal (i.p.) LPS challenge of *Clec1b^−/−^* and *Clec1b^+/+^* reconstituted animals (Fig. 2A–C and Supporting Information Fig. 5). It has been shown that B lymphocyte stimulation via LPS/TLR4 favors their emigration from the blood into SLOs 42. Interestingly, we could not observe any CLEC‐2^pos^ B lymphocytes in the SLOs of LPS‐stimulated mice (Fig. 2), supporting the observation that circulating activated B lymphocytes downregulate CLEC‐2 before entering into SLOs (Fig. 1B). Kerrigan and collaborators have suggested the same CLEC‐2 downregulation process by circulating CD11b^high^ Gr‐1^high^ cells upon reaching inflammatory sites 19. As entry to both SLOs and inflammatory sites requires leucocyte rolling, arrest, and transendothelial migration 41, it is tempting to suggest that CLEC‐2 downregulation or shedding by these leucocytes facilitates the completion of this three‐step mechanism. Indeed, it has been shown that the shedding of transmembrane molecules is essential for leucocyte transendothelial migration 43, 44, 45, 46. We hypothesize that CLEC‐2 could be lost via the same mechanisms.

CD11b^high^ Gr‐1^high^ cells, pDCs, and CD11b^neg/int^ cDCs isolated from LPS‐stimulated *Clec1b^−/−^* mice exhibited a higher level of 17D9 binding than *Clec1b^−/−^* nonchallenged counterparts. Once again, these results indicate that the 17D9 antibody clone has the capacity to bind LPS‐stimulated leucocytes in a CLEC‐2‐independent manner. The lack of *Clec1b* transcript upregulation in LPS‐stimulated leucocytes (Fig. 2E) provides further evidence to support this conclusion and challenges previously described LPS‐induced CLEC‐2 upregulation by most splenic resident leucocytes and peripheral LN cDCs 23, 24.

### MLN but not splenic CD11b^high^ cDCs acquire CLEC‐2 following LPS stimulation

In agreement with the results detailed above, CLEC‐2 expression was not detected on splenic CD11b^high^ cDCs. However, a modest but significant increase in staining of MLN‐derived *Clec1b^+/+^* CD11b^high^ cDCs compared to *Clec1b^−/−^* controls was observed after LPS injection (Fig. 2D), indicating that LPS‐stimulated MLN CD11b^high^ cDCs have the capacity to upregulate CLEC‐2 (Fig. 2D). However, we could not correlate the appearance of CLEC‐2 on the membrane with a higher relative amount of *Clec1b* transcripts in the stimulated CD11b^high^ cDCs (Fig. 2E), suggesting that LPS stimulation regulates CLEC‐2 expression in MLN CD11b^high^ cDCs via posttranscriptional mechanisms.

Taken together, our results confirmed high levels of CLEC‐2 expression on splenic platelets (data not shown), while no significant expression of CLEC‐2 was observed on most leucocyte populations investigated, both at steady state and after LPS injection. However, we did observe an increase in CLEC‐2 expression on activated CD11b^high^ cDCs isolated from the MLN. This increase was absent on splenic‐activated cDCs.

The existence of cell‐specific and tissue‐specific regulation of CLEC‐2 expression has previously been observed in the context of human rheumatoid arthritis, a chronic inflammatory disease where CLEC‐2 expression was found to be restricted to tissue infiltrating platelets, while absent from activated DCs 22. In contrast, FITC skin painting, FITC footpad immunization, or OVA/CFA subcutaneous injections in mice were found to contribute to the generation of an immune response that relies on CLEC‐2 expression by activated DCs and their interaction with PDPN^pos^ lymphatic endothelial cells and PDPN^pos^ FRCs 14, 15, 24. These studies demonstrate that CLEC‐2 upregulation is a characteristic of locally activated DCs migrating toward the draining LN 14, 15, 24 and not the systemic feature of an activated immune system 23. In accordance with this, we only observed CLEC‐2 upregulation by activated DCs in the MLN, a SLO close to the LPS site of administration (i.e. the peritoneal cavity), but not in a remote SLO such as the spleen. This suggests that the MLN CLEC‐2^pos^ CD11b^high^ cDCs we observed may have recently migrated from the surrounding mesenteric tissue following LPS administration.

### Normal lymphocyte homeostasis in B cell *Clec1b* ^−/−^ mice as B cells do not produce CLEC‐2

**Figure 3:**
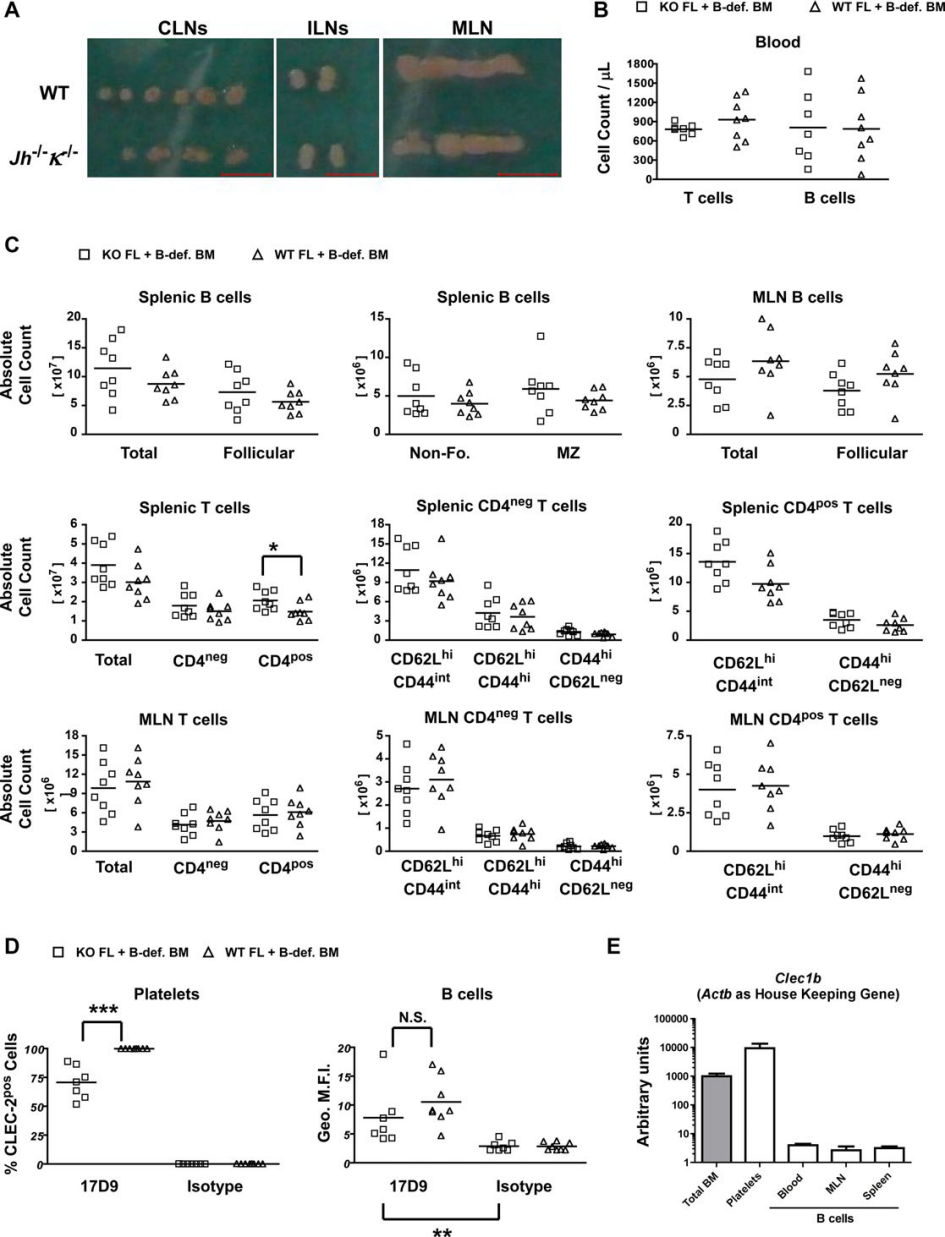
B lymphocyte‐specific *Clec1b* deficiency does not affect lymphocyte homeostasis as CLEC‐2 molecules on circulating B cells are exogenously derived. (A) Cervical lymph nodes (*CLNs*), inguinal lymph nodes (*ILNs*), and *MLN* were isolated from *WT* and B‐cell deficient *Jh* ^−/−^ *κ* ^−/−^ mice and imaged. The picture is representative of five WT and six B‐cell deficient mice. Red bars represent 5 mm. (B–D) Irradiated recipient mice were reconstituted with a mix of 2 × 10^5^ *Clec1b^+/+^* or *Clec1b^−/−^* E14.5 FL cells and 18 × 10^5^ *Jh* ^−/−^ *κ* ^−/−^ BM cells. Seven to nine weeks later, the blood, spleen, and MLNs were isolated and analyzed by flow cytometry to determine (B and C) leucocyte numbers and (D) CLEC‐2 expression was assessed using the 17D9 antibody compared to its respective isotype control. Each symbol represents an individual mouse and bars represent means. The graphs summarize two independent experiments pooled together. (E) Platelets and B lymphocytes were isolated from the blood, MLN, and the spleen of C57BL/6 mice by FACS, based on the following phenotypes: platelets: FSC^low^ CD41^pos^; B cells: FSC^hi^ CD41^neg^ DAPI^neg^ CD3ε^neg^ CD19^pos^_._ After mRNA isolation and cDNA synthesis, the relative expression of *Clec1b* transcripts was analyzed by quantitative PCR. The signals for *Clec1b* was normalized against the house‐keeping gene *Actnb* (β‐actin). *Total BM* was used as positive control and reference to set the arbitrary unit. Each population was isolated from three independent cell sorting experiments, including one to two mice for each cell sorting, and one quantitative PCR was ran from each cell sorting. The graphs summarize these three independent quantitative PCRs pooled together and data are shown as mean + SEM. Statistical significance was measured by a Mann–Whitney test with a 95% confidence interval where: *p < 0.05; **p < 0.005; ***p < 0.0005; N.S, not significant.

To gain insight into the potential physiological roles of CLEC‐2 in B lymphocytes, we first visualized the LNs from B lymphocyte‐deficient *Jh* ^−/−^ *κ* ^−/−^ mice and saw no evidence of erythrocyte infiltration in the LNs, similar to WT animals (Fig. 3A), indicating that CLEC‐2 on B lymphocytes does not play an important role in blood–lymph separation or the maintenance of HEVs.

In order to investigate if CLEC‐2 on peripheral blood B lymphocytes contributes to lymphocyte homeostasis, we generated mice with a *Clec1b* ^−/−^ deficiency restricted to the B‐cell lineage by mixing *Clec1b* ^−/−^ FL and *Jh* ^−/−^ *κ* ^−/−^ BM cell suspensions (at a 1:9 ratio) that we injected into lethally irradiated C57BL/6 recipients (Supporting Information Fig. 7A) 47. The absolute numbers of lymphocytes in the blood, spleen, and MLNs were monitored (Fig. 3B and C, Supporting Information Fig. 7B and C). Both in the blood and the SLOs, the absolute numbers of B and T lymphocytes were normal. In the spleen, the numbers of follicular, nonfollicular, and marginal zone *Clec1b* ^−/−^ B lymphocytes were comparable to the controls. Contrary to the MLNs, a small increase in CD4^+^ T lymphocytes was observed in the spleen of *Clec1b* ^−/−^ animals. However, no‐significant increase in naïve (CD62L^hi^ CD44^int^) or activated (CD44^hi^ CD62L^−^) CD4^+^ T lymphocytes was noted in these animals. Similarly, the CD4^−^ T lymphocytes showed the same level of activation between *Clec1b* ^−/−^ and control animals. These results indicate that deletion of the *Clec1b* gene in the B‐cell lineage has no effect on lymphocyte homeostasis.

Despite the absence of a functional *Clec1b* gene, peripheral blood *Clec1b* ^−/−^ B lymphocytes were stained by 17D9 at the same level as *Clec1b* ^+/+^ B lymphocytes (Fig. 3D). In B lymphocyte *Clec1b*‐deficient mice, 71% of the platelets were CLEC‐2^pos^ on average (Fig. 3D), while the level of CLEC‐2 expression by CD11b^high^ Gr‐1^high^ cells was comparable to that found in controls (Supporting Information Fig. 7D). We compared the amount of *Clec1b* transcripts by quantitative PCR in B lymphocytes isolated from the peripheral blood, MLNs, and spleen. In all three populations, the amount of *Clec1b* was extremely low and at a comparable level (Fig. 3E). This last result corroborates a study on rat B lymphocytes showing that *Clec1b* transcripts are hardly detectable in these cells 48. From these observations, we conclude that peripheral blood B lymphocytes do not intrinsically express CLEC‐2. Instead we propose that the CLEC‐2 molecules detected on the surface of circulating B lymphocytes are derived from MHC‐II antigen presentation, trogocytosis, or exosomes/microparticles attached to the B‐cell membrane 49.

### Concluding remarks

Our study confirms that CLEC‐2 is constitutively expressed by mouse platelets and circulating CD11b^high^ Gr‐1^high^ myeloid cells and shows for the first time that CLEC‐2 is present on the surface of peripheral blood B lymphocytes. These B cells do not produce CLEC‐2 but likely acquire CLEC‐2 molecules from other yet uncharacterized cell types. Our data suggest that both circulating B lymphocytes and CD11b^high^ Gr‐1^high^ myeloid cells lose CLEC‐2 when entering SLOs. This loss of CLEC‐2 might depend on the same mechanisms that are selectively shedding CD23 and CD62L from leucocytes during transendothelial migration 43, 45, 46. As CLEC‐2 stimulates PDPN^pos^ FRCs in SLOs in order to mount a proper immune response 14, 15, 24, we propose that the loss of CLEC‐2 by naïve B lymphocytes and CD11b^high^ Gr1^high^ myeloid cells entering in the SLOs might be a prerequisite to prevent untimely FRCs activation in absence of antigenic challenge.

The use of animals reconstituted with *Clec1b* ^−/−^ FL and the measurements of *Clec1b* transcripts allowed us to rule out any constitutive CLEC‐2 expression by most of the leucocyte subpopulations isolated from SLOs. We also demonstrated that isotype controls are not adequate when working with the 17D9 antibody clone. Finally, we showed that LPS peritoneal injection induces CLEC‐2 acquisition to the unique MLN‐activated DCs leucocyte population. Taken together with other studies, our findings emphasize the notion that the expression of CLEC‐2 is not only restricted to specific subsets of resting leucocytes and platelets but is determined both by the activation state and anatomical site where immune responses take place.

## Materials and methods

### Mice and diets

*Clec1b* ^+/−^ 25, *Clec1b* ^fl/fl^ 25, *Rosa26* ^+/ERT2cre^ (Jackson Laboratory, ME) 50, *Clec1b* ^fl/fl^ x *Rosa26* ^+/ERT2cre^, *Jh* ^−/−^ *κ* ^−/−^ 51, 52, and BoyJ mice were maintained in the Biomedical Services Unit, University of Birmingham. C57BL/6 mice were purchased from Harlan, UK. Animals were fed with FormulaLab Diet 5008 (LabDiet, St‐Louis, MO). When required, 6‐ to 8‐week‐old *Clec1b* ^fl/fl^ × *Rosa26* ^+/ERT2cre^ and their *Clec1b* ^fl/fl^ x *Rosa26* ^+/+^ control littermates were continuously fed with tamoxifen‐supplemented diet TAM 400 (Harlan, UK). For isolation of embryonic FL, the morning of vaginal plug detection was designated day 0.5 of gestation. Animal experiments were performed in accordance with UK Home Office legislation.

### Mouse hematopoietic system reconstitution

C57BL/6 or BoyJ mice (8–10 weeks old) were given Baytril in the drinking water for 7 days prior to irradiations with two doses of 450rad, 3 h apart. One hour after the last irradiation *Clec1b^+/+^* or *Clec1b^−/−^* E14.5 FL cells were injected intravenously (i.v.). For the generation of B‐cell *Clec1b*‐deficient recipients, 2 × 10^5^ *Clec1b^+/+^* or *Clec1b^−/−^* E14.5 FL cells were mixed with 18 × 10^5^ *Jh* ^−/−^ *κ* ^−/−^ BM cells. Mice were left for 6‐ to 8‐weeks postinjection before analysis or further challenged by i.p. injection of 25 μg of LPS (Chondrex) diluted in PBS or PBS only. Mice were analyzed 16–18 h post LPS or PBS injection. Successful LPS injections were confirmed by ≥5% weight loss over this period.

### Tissue sampling and preparation

In all cases, cell centrifugation was performed at the average force of 275 g for 4 min. Blood was sampled from the tail vein of *Clec1b^fl/fl^*; *Rosa26^+/ERT2cre^*; or *Clec1b^fl/fl^* × *Rosa26^+/ERT2cre^* mice into 20 mM EDTA/PBS 1× solution. After centrifugation, the supernatant was removed and the pellet resuspended in red blood cell lysis buffer (Sigma‐Aldrich) at room temperature for 5 min. Samples were centrifuged and resuspended in cold PBS 1×, 2% foetal calf serum (FCS), 2 mM EDTA solution and stained for flow cytometry analysis or processed for genomic DNA extraction.

Whole blood from FL reconstituted animals was drawn into acid citrate dextrose solution (9:1 v/v) from the inferior vena cava under isofluorane anesthesia and mixed to 20 mM EDTA/PBS 1× solution. An aliquot of blood was centrifuged and processed as described above.

The spleen and the MLN were harvested into cold 2% FCS RPMI solution (Sigma‐Aldrich). For cell sorting of T cells, B cells, and NK cells, part of the spleen was mechanically dissociated on a 100 mm mesh (Greiner Bio‐one). In all the other cases, spleen or MLN were teased apart with dissection forceps in a 2% FCS RPMI solution containing 2.5 mg/mL of Collagenase D (Roche) and 2 mg/mL of DnaseI (Sigma‐Aldrich). Cell suspensions were kept under magnetic stirring at 37°C for 45 min before centrifugation. Pellets were resuspended in a 2% FCS RPMI solution containing 2.5 mg/mL of Collagenase/Dispase (Roche) and 2 mg/mL of DnaseI (Sigma‐Aldrich) and kept under magnetic stirring at 37°C for 30 min. Cell suspensions were adjusted to a final EDTA concentration of 5 mM by the addition of a 0.5 M EDTA solution and kept under magnetic stirring at 37°C for 5 min. Cell suspensions were centrifuged and the pellets resuspended in red blood cell lysis buffer (Sigma‐Aldrich) as described above before processing for FACS staining.

### Antibodies, FACS analysis, and cell sorting

The full list of antibodies used is provided in Supporting Information Table 1. Anti‐mouse CLEC‐2‐FITC 17D9 clone was mainly obtained from a commercial provider (AbD Serotec, 17D9) and compared to rat IgG2b‐FITC (AbD Serotec, MCA1125FT). For some experiments, purified 17D9 (a kind gift from Caetano Reis e Sousa, Cancer Research UK, London) and purified rat IgG2b (R&D Systems) were used. Purified anti‐mouse CLEC‐2 INU1 clone (a kind gift from Bernhard Nieswandt, University of Würzburg, Germany) was used in conjunction with purified rat IgG1k (Biolegend, 400402). All purified antibodies were conjugated to AlexaFluor®488 using a monoclonal antibody labeling kit (Invitrogen).

Cells were stained with antibodies at 4°C in the dark in PBS 1×, 2% FCS, 2 mM EDTA solution. Cells were washed and centrifuged twice before being resuspended in a cold a PBS 1×, 2% FCS, 2 mM EDTA, 1 mg/mL DAPI solution. Flow cytometry acquisitions were performed on a three laser (405, 488, 633 nm) Cyan (Beckman Coulter) using Summit v4.3 software (Beckman Coulter). Flow cytometry cell sorting was performed on a MoFlo high‐speed cell sorter and Astrios cell sorter (Beckman Coulter) using Summit software (Beckman Coulter). FACS data were analyzed with FlowJo software 8.7 (Tristar).

### Quantitative PCR

Total BM was flushed with 2% FCS RPMI from the tibia and femur of both hind legs. A tenth of the cell suspension was centrifuged, the supernatant was removed and pellets were snap frozen on dry ice. Flow cytometry cell sorted cells were collected in PBS 1×, 2% FCS, 2 mM EDTA solution, centrifuged, pelleted, snap frozen on dry ice and stored at −80°C. mRNA was extracted using a “RNeasy Microkit” (Qiagen) following the manufacturer's instructions. cDNA was generated using the “High Capacity Reverse Transcription” kit and random primers mix (Applied Biosystems). RT‐PCR reaction settings were: 25°C, 10 min; 37°C, 120 min; 85°C, 10 min. The cDNA obtained was diluted 1:2 with RNase‐free/DNase‐free water (Qiagen), preamplified using the “TaqMan Pre‐Amp Master Mix” kit (Applied Biosystems) and the TaqMan probes (Applied Biosystems) specific for murine β‐actin (*Actnb*, probe number: Mm_01205647‐g1), β‐2microglobulin (*B2m*, probe number: Mm_00437762‐m1) and CLEC‐2 (*Clec1b*, probe number: Mm_0183353‐m1). The preamplification PCR reaction settings were 95°C, 10 min followed by ten cycles: (95°C, 15 s; 65°C, 4 min). These preamplified cDNA were diluted 1:5 with RNase‐free/DNase‐free water (Qiagen). The TaqMan probe‐based quantitative PCR was set up on a 384 well/plate using “TaqMan Gene Expression Master Mix”(Applied Biosystems) and the TaqMan probes for the murin *Actnb, B2m*, and *Clec1b* mentioned above. The quantitative PCR reactions were run on a 7900HT quantitative PCR machine (Applied Biosystems) at 50°C, 2 min; 95°C, 10 min; 40 cycles: (95°C, 15 s; 60°C, 1 min).

### Statistical analysis

All statistical analyses were performed on Prism v4.0 (GraphPad Software, CA) using two‐tailed Mann–Whitney tests with 95% confidence interval.

## Conflict of interest

The authors declare no commercial or financial conflict of interest.

**Abbreviations**
BMDCs: bone marrow‐derived dendritic cells
cDCs: conventional dendritic cells
Clec1b: C‐type lectin domain family 1, member B
CLEC‐2: C‐type lectin‐like receptor 2
FL: foetal liver
FRCs: fibroblastic reticular cells
HEVs: high endothelial venules
pDCs: plasmacytoid dendritic cells
PDPN: podoplanin
SLOs: secondary lymphoid organs

## Supporting information

As a service to our authors and readers, this journal provides supporting information supplied by the authors. Such materials are peer reviewed and may be re‐organized for online delivery, but are not copy‐edited or typeset. Technical support issues arising from supporting information (other than missing files) should be addressed to the authors.

Supporting information: Click here for additional data file.

Peer review correspondence
