# Supplementary material for: The expression of mouse CLEC‐2 on leucocyte subsets varies according to their anatomical location and inflammatory state
Source: Eur J Immunol. 2015 Aug 12;45(9):2484–93. doi: 10.1002/eji.201445314 (PMC4737233; doi:10.1002/eji.201445314)
Supplement: Supplementary file 1 — Supporting information: [file EJI-45-2484-s001.pdf]

European Journal of Immunology

Supporting Information
for
DOI 10.1002/eji.201445314

Kate L. Lowe, Leyre Navarro-Núñez, Cécile Bénézech, Saba Nayar, Bethany L. Kingston, Bernhard Nieswandt, Francesca Barone, Steve P. Watson, Christopher D. Buckley and Guillaume E. Desanti

The expression of mouse CLEC-2 on leucocyte subsets varies according to their anatomical location and inflammatory state

© 2015 The Authors. *European Journal of Immunology* published by WILEY-VCH Verlag GmbH & Co. KGaA, Weinheim.

Supporting information:

The expression of mouse CLEC-2 on leucocyte subsets varies according to their anatomical location and inflammatory state

Kate L. Lowe[1], Leyre Navarro-Nuñez[1], Cécile Bénézech[2*], Saba Nayar[3], Bethany L. Kingston[3,4], Bernhard Nieswandt[5], Francesca Barone[3], Steve P. Watson[1], Christopher D. Buckley[3], Guillaume E. Desanti[3,#]

Supporting Information Figure 1: *Clec1b* excision is induced by 6 month of tamoxifen supplemented diet in the *Clec1b$^{fl/fl}$xRosa26$^{+/creERT2}$* **mouse line.** (A) The *Clec1b* locus was modified by introducing LoxP sites between exons 3 and 4, creating a floxed *Clec1b* allele (*Clec1b$^{fl/fl}$, Conditional Clec1b locus*). *Clec1b$^{fl/fl}$* mice were crossed to mice expressing the fusion protein Cre-recombinase/estrogen receptor T2 (*CreERT2*) under the control of the *Rosa26* promoter and the adenovirus splice receptor (*AdenoSA*). The ERT2 moiety sequesters Cre-recombinase in the cytoplasm until administration of tamoxifen which allows Cre translocation to the nucleus to excise the floxed *Clec1b* allele (*KO Clec1b locus*). The excision of exons 3 and 4 induces a frame shift to exons 5 and 6 abrogating transcription and therefore inhibiting CLEC-2 expression. (B) Genomic PCR on erythrocyte-depleted peripheral blood from *Clec1b$^{fl/fl}$xRosa26$^{+/creERT2}$* (*Clec1b$^{fl/fl}$ CreERT2*) and *Clec1b$^{fl/fl}$* controls fed tamoxifen-supplemented diet for 6 months. *Clec1b$^{fl/fl}$xRosa26$^{+/creERT2}$* mice (11-14 weeks old) fed with conventional diet only were used as controls. The presence of the wild-type *Clec1b* locus and the floxed *Clec1b* locus can be detected at 230bp and 333bp, respectively (*Gel Clec1b WT*). The excised *Clec1b* locus is detected at 344bp (*Gel Clec1b KO*) by an independent genomic PCR reaction. The following primer sequences were used:
Clec1b wild-type forward, GATGAGTCTGCTAGGGATGC;
Clec1b knockout forward, CAGAGGAAGAAAACTCAGAAGG;
Clec1b common reverse, AGCCTGGAGTAACAAGATGG.
The PCR reaction protocols were: 95°C, 5 min; 35 cycles: [95°C, 30 sec; 60°C, 30 sec; 72°C, 1 min]; 72°C, 10 min; 4°C. Results shown are representative of 4-13 mice.

Supporting Information Figure 2: CLEC-2 is present at the surfaces of peripheral blood platelets, B cells and CD11b^{high} Gr-1^{high} cells at steady-state. Flow cytometry staining strategy to identify the haematopoietic cell subsets found in peripheral blood. Platelets and leucocytes were gated based on their small size (low FSC) and expression of CD41; or larger size (higher FSC) and absence of CD41, respectively. DAPI^{Pos} dead cells were excluded from the analysis. B and T lymphocytes (*Lympho*) and myeloid cells were gated based on their relative expression of CD3ε and CD19. Myeloid subsets were further separated based on their expression of CD11b and Gr-1. Cells positive for CLEC-2 are represented on histograms as a percentage of total cell counts in the population.

This analysis shows results from *Clec1b*$^{+/+}$ or *Clec1b*$^{-/-}$ foetal liver reconstituted animals (WT FL or KO FL, respectively) challenged by intraperitoneal injection of 25μg LPS or PBS (Control). CLEC-2 expression was detected using the 17D9 antibody compared to its respective isotype control. This gating strategy was also applied to peripheral blood samples from tamoxifen-treated *Clec1b*$^{fl/fl}$*xRosa26*$^{+/creERT2}$ and *Clec1b*$^{fl/fl}$ mice and when analysing CLEC-2 expression with INU1.

Supporting Information Figure 3: Circulating T-cells do not express CLEC-2. (A) *Clec1b*$^{fl/fl}$*xRosa26*$^{+/creERT2}$ mice (*CRE TAM*) and *Clec1b*$^{fl/fl}$ control mice (*LOX TAM*) were fed tamoxifen-supplemented diet from 6-8 weeks old for 6 months. *Clec1b*$^{fl/fl}$*xRosa26*$^{+/creERT2}$ mice (11-14 weeks old) fed conventional diet only were used has controls (*CRE Control*). Blood was drawn from the tail vein, the erythrocytes were lysed the remaining leucocytes stained. CLEC-2 expression was assessed using the 17D9 (*white symbols*) or INU1 (*black symbols*) antibody clones and compared to their respective isotype controls by flow cytometry. The staining intensities are expressed as the geometric mean of fluorescence intensity (*Geo.M.F.I*). (B) Wild-type animals were lethally irradiated and injected intravenously with *Clec1b*$^{+/+}$ or *Clec1b*$^{-/-}$ E14.5 foetal liver cells (WT FL or KO FL, respectively). 6-8 weeks post foetal liver injection, a cohort of animals was challenged with 25μg of LPS by intraperitoneal injection (*black symbols*) and compared to non-challenged animals (*white symbols*). 16-18 hours post LPS injection, blood samples were obtained, processed and analysed as described above. Each symbol represents a sample from an individual mouse. Bars represent the means. The graphs summarize 1-3 independent experiments pooled together. Statistical significance was measured by a Mann-Whitney test

with a 95% confidence interval where; *: $P<0.05$; **: $P<0.005$; ***: $P<0.0005$; N.S.: not significant.

Supporting Information Figure 4: Flow cytometry staining strategy to identify the haematopoietic cell subsets found in the spleen. Platelets and leucocytes are gated based on their small size (low FSC) and expression of CD41; or larger size (higher FSC) and absence of CD41, respectively. Leucocyte populations are positively selected with CD45. Myeloid subsets are separated based on their expression of CD11b and Gr-1. B lymphocytes (B Lympho.) are defined as $B220^{pos}$ $CD11b^{neg}$. Plasmacytoid dendritic cells (p-DC) are defined as $B220^{pos}$ $CD11c^{pos}$ $Gr\text{-}1^{pos}$. $CD11c^{pos}$ $B220^{neg}$ conventional dendritic cells (c-DC) are separated into two subsets based on their CD11b expression level. CLEC-2 expression is detected using the 17D9 antibody compared to its respective isotype control. Cells positive for CLEC-2 are represented on histograms as a percentage of total cell counts in the population. This analysis shows results from *Clec1b*$^{+/+}$ or *Clec1b*$^{-/-}$ foetal liver reconstituted animals (WT FL or KO FL, respectively) that were challenged by intraperitoneal injection with 25µg LPS or PBS only (Control). This gating strategy was also applied to analyse cells from the MLN.

Supporting Information Figure 5: Plasmacytoid DC from the spleen and the mesenteric lymph node (MLN) do not express CLEC-2 at steady-state or after LPS injection.. *Clec1b*$^{+/+}$ or *Clec1b*$^{-/-}$ foetal liver chimeras (WT FL or KO FL, respectively) at 6-8 weeks old were challenged by intraperitoneal injection with 25µg LPS (*black symbols*) and compared to non-challenged animals (*white symbols*). 16-18 hours post injection, the spleen and MLN were harvested, the erythrocytes were lysed and the remaining leucocytes stained. CLEC-2 expression was addressed using the 17D9 antibody compared to its respective isotype control by flow cytometry. The staining intensities are expressed by the geometric mean of fluorescence intensity (*Geo.M.F.I*). Each symbol represents a sample from an individual mouse. The graphs summarize 3 independent experiments pooled together. Statistical significance was measured by a Mann-Whitney test with a 95% confidence interval where: *: $P<0.05$; **: $P<0.005$; ***: $P<0.0005$; N.S.: not significant.

Supporting Information Figure 6: Publicly available microarray data analysis for *Clec1b* expression in several organs and in multiple subsets of mouse leucocytes isolated from different tissues. (A) Data obtained online from the ImmGen consortium (http://www.immgen.org/). (B) Data obtained online from the BioGPS consortium (http://www.biogps.org).

Supporting Information Figure 7: B lymphocyte-specific *Clec1b*-deficiency does not affect lymphocyte homeostasis and CLEC-2 expression by CD11b^{high} Gr-1^{high} cells. Mice with a B lymphocyte-specific *Clec1b*-deficiency were generated by injecting 2x10^{5} *Clec1b*$^{-/-}$ E14.5 foetal liver cells mixed with 18x10^{5} *Jh*$^{-/-}$*κ*$^{-/-}$ bone marrow cells into irradiated C57BL/6 hosts. Controls were generated by injecting a mix of 2x10^{5} *Clec1b*$^{+/+}$ E14.5 foetal liver cells with 18x10^{5} *Jh*$^{-/-}$ *κ*$^{-/-}$ bone marrow cells. 7-9 weeks after the injections, the reconstituted mice were studied (A). (B-C) Flow cytometry staining strategy to identify the haematopoietic cell subsets found in the spleen. B-lymphocytes are defined as B220^{pos} and subsequently sub-divided into the CD21^{neg} CD23^{neg} IgM^{high} IgD^{neg} non-follicular B-cells; CD21^{hi} CD23^{int} IgM^{high} IgD^{int} marginal zone B-cells; or CD23^{hi} CD21^{int} IgM^{int} IgD^{high} follicular B-cells (B). T-lymphocytes are defined as CD3ε^{pos} B220^{neg} and subsequently sub-divided into CD4^{neg} T-cells and CD4^{pos} T-cells. In these two T-cell populations, the naïve cells are CD62L^{pos} CD44^{int} while the activated cells are CD44^{hi} CD62L^{neg} (C). CLEC-2 expression was assessed on peripheral blood CD11b^{high} Gr-1^{high} cells using the 17D9 antibody compared to its respective isotype control. The staining intensities are expressed by the geometric mean of fluorescence intensity (*Geo.M.F.I*). Each symbol represents a sample from an individual mouse either reconstituted with *Clec1b*$^{-/-}$ foetal liver (*open squares*) or with *Clec1b*$^{+/+}$ foetal liver (*open triangles*) mixed with *Jh*$^{-/-}$ *κ*$^{-/-}$ bone marrow cells (D). Results summarize 2 independent experiments pooled together. Statistical significance was measured by a Mann-Whitney test with a 95% confidence interval where: *: P<0.05; **: P<0.005; ***: P<0.0005; N.S.: not significant. Abbreviations: B-def: B-lymphocyte deficient.

Supporting Information Table 1: List of antibodies used for the flow cytometry analysis.

Supporting Information Figure 1:

Kate L. Lowe et al. (2015)

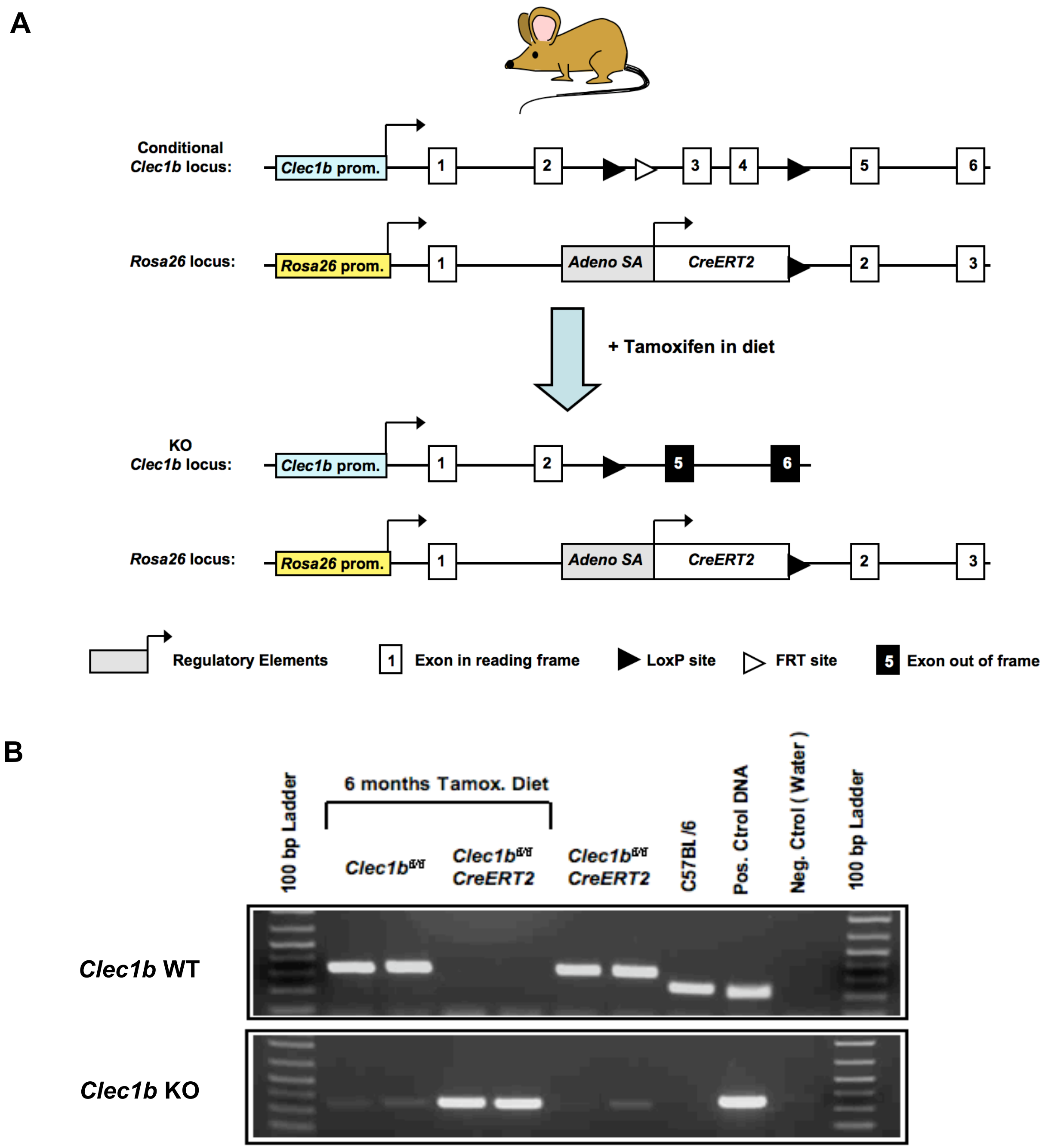

Supporting Information Figure 2:

Kate L. Lowe et al. (2015)

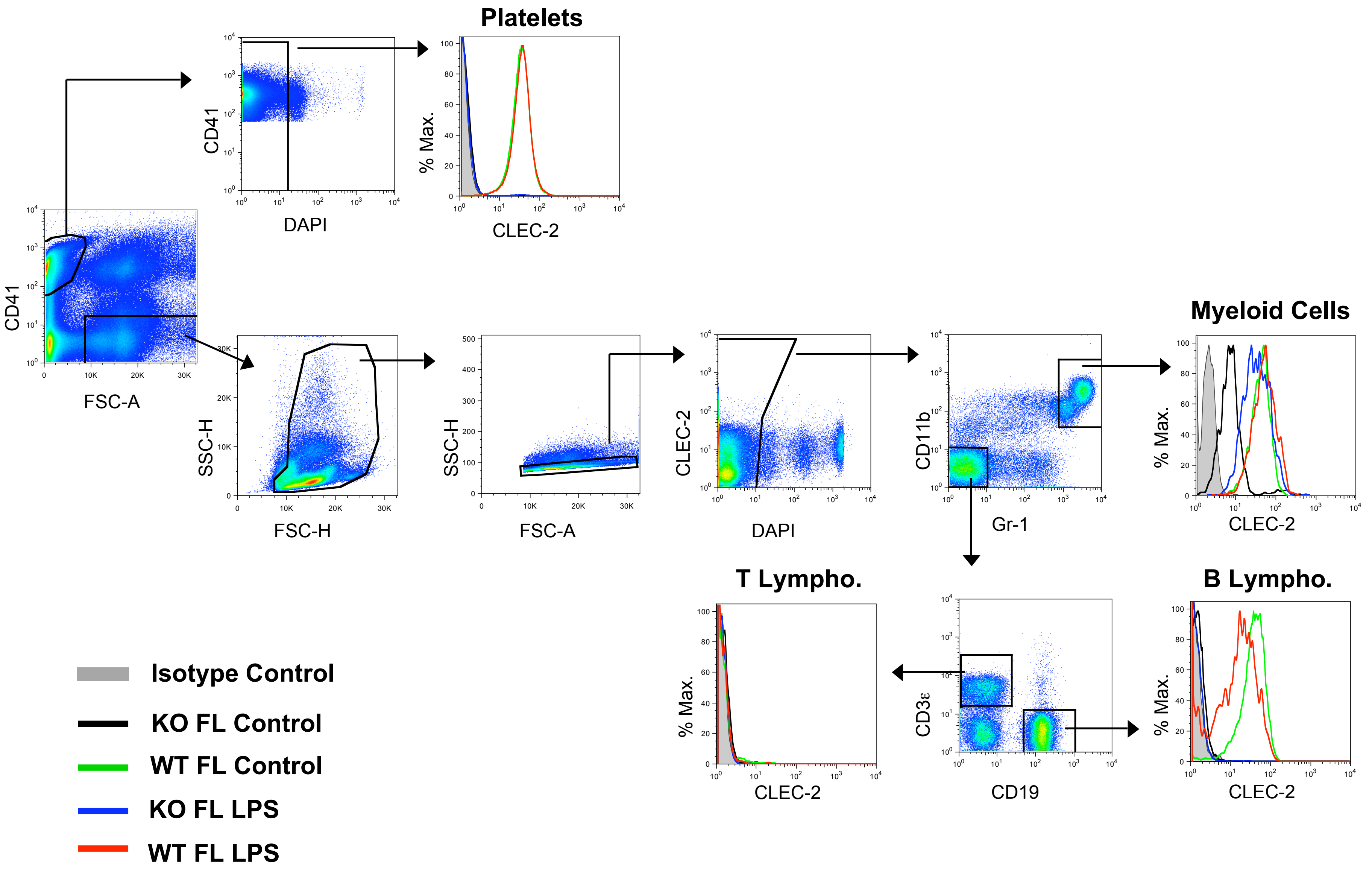

Supporting Information Figure 3:

Kate L. Lowe et al. (2015)

A

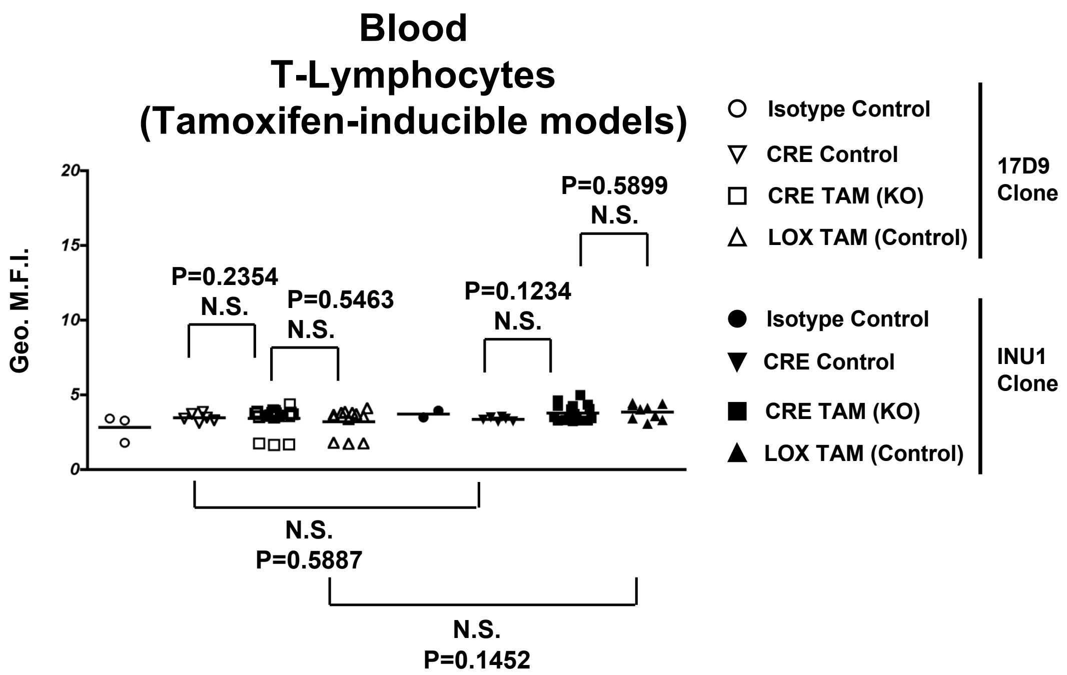

B

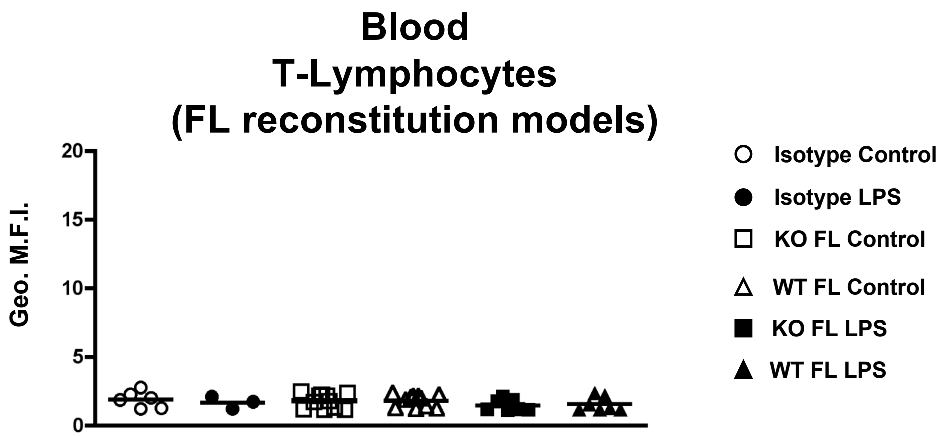

Supporting Information Figure 4: Kate L. Lowe et al. (2015)

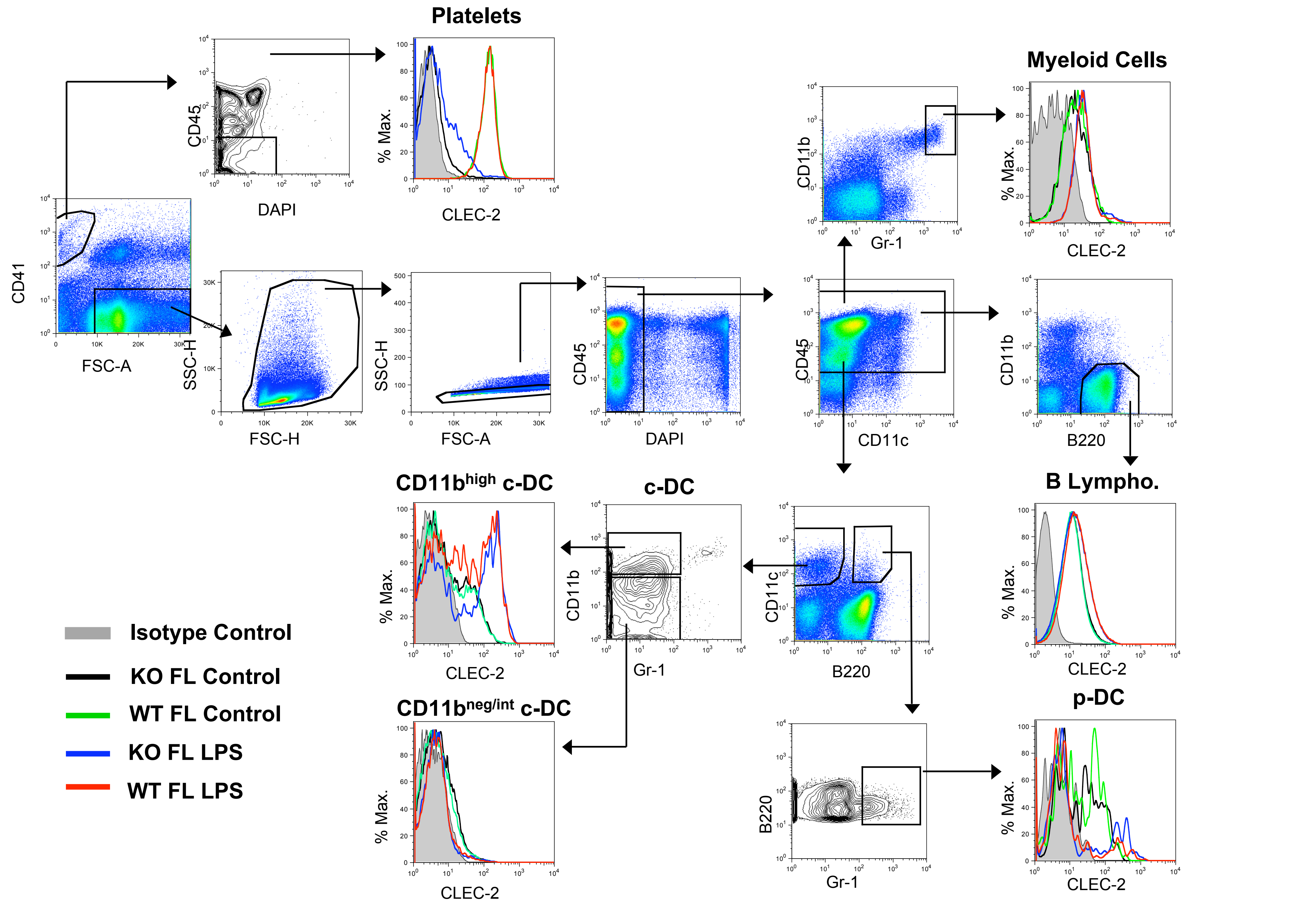

Supporting Information Figure 5:

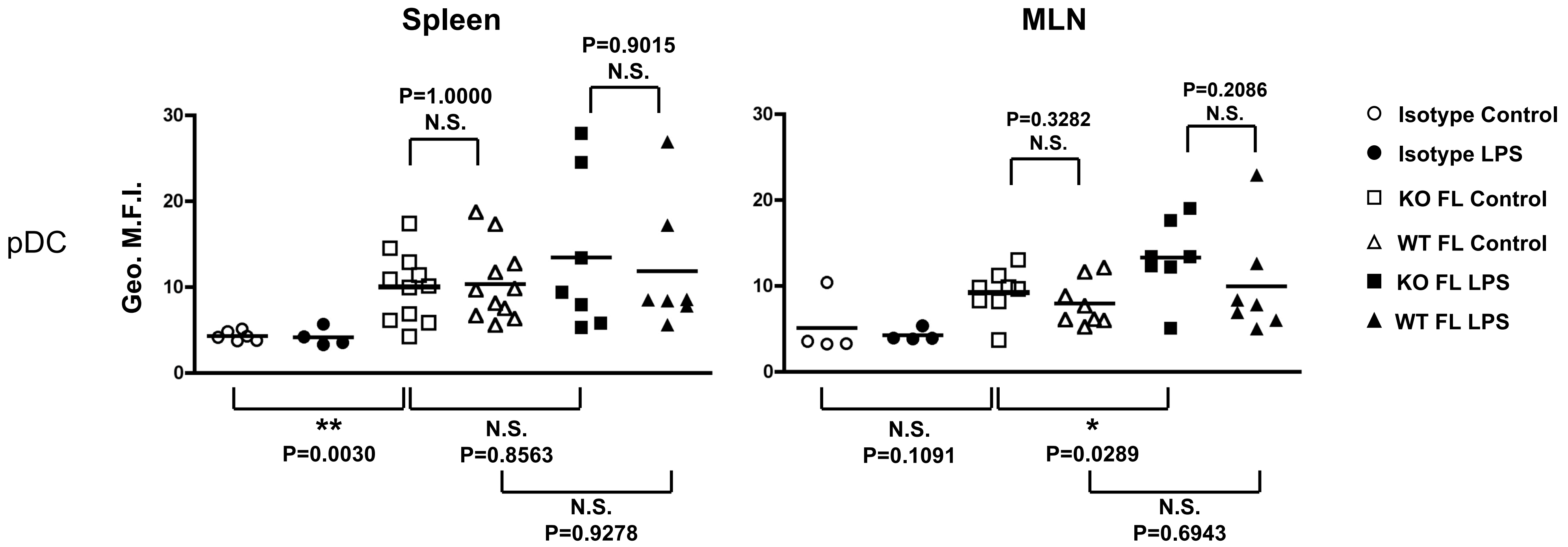

Supporting Information Figure 6:

A

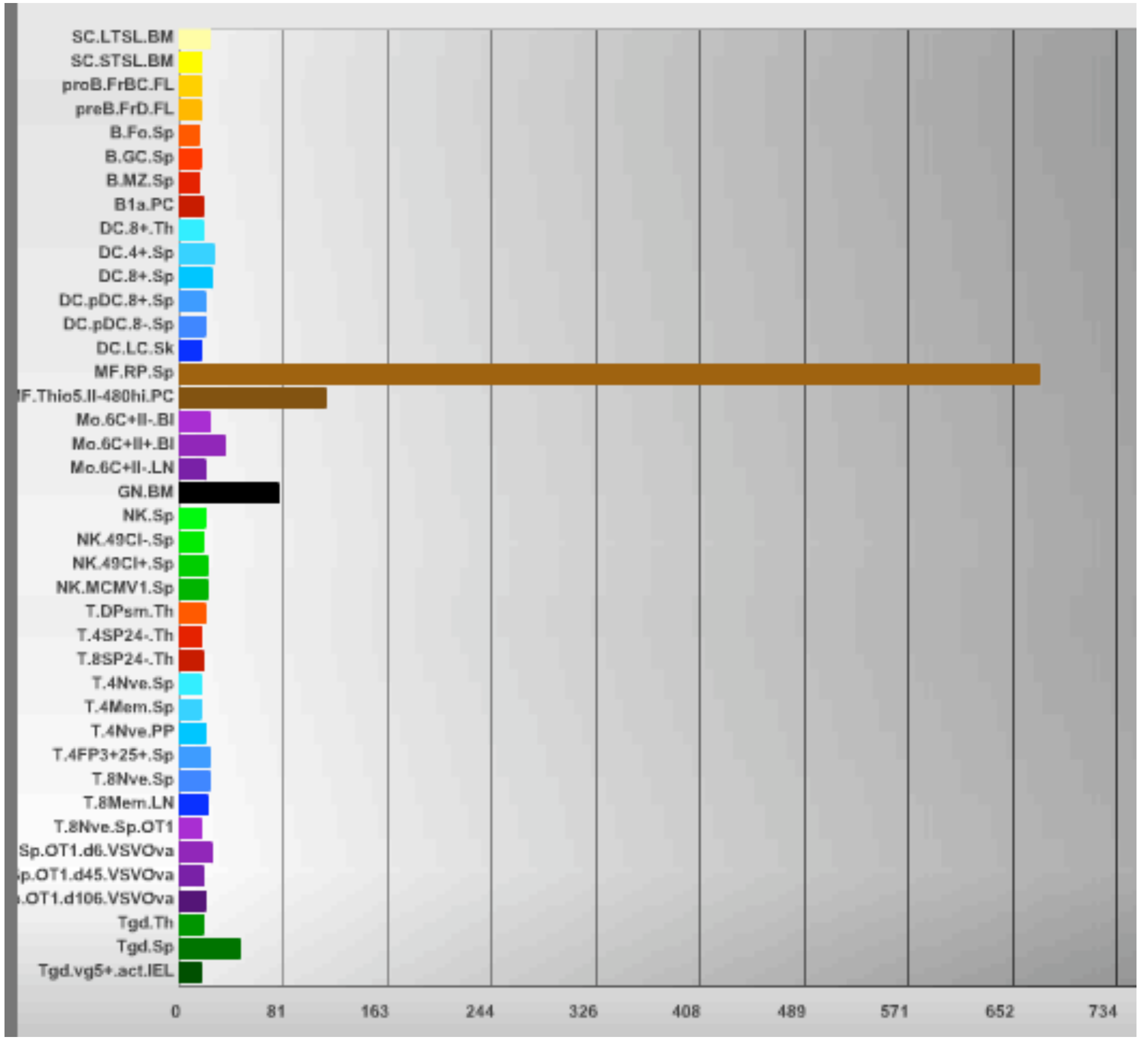

B

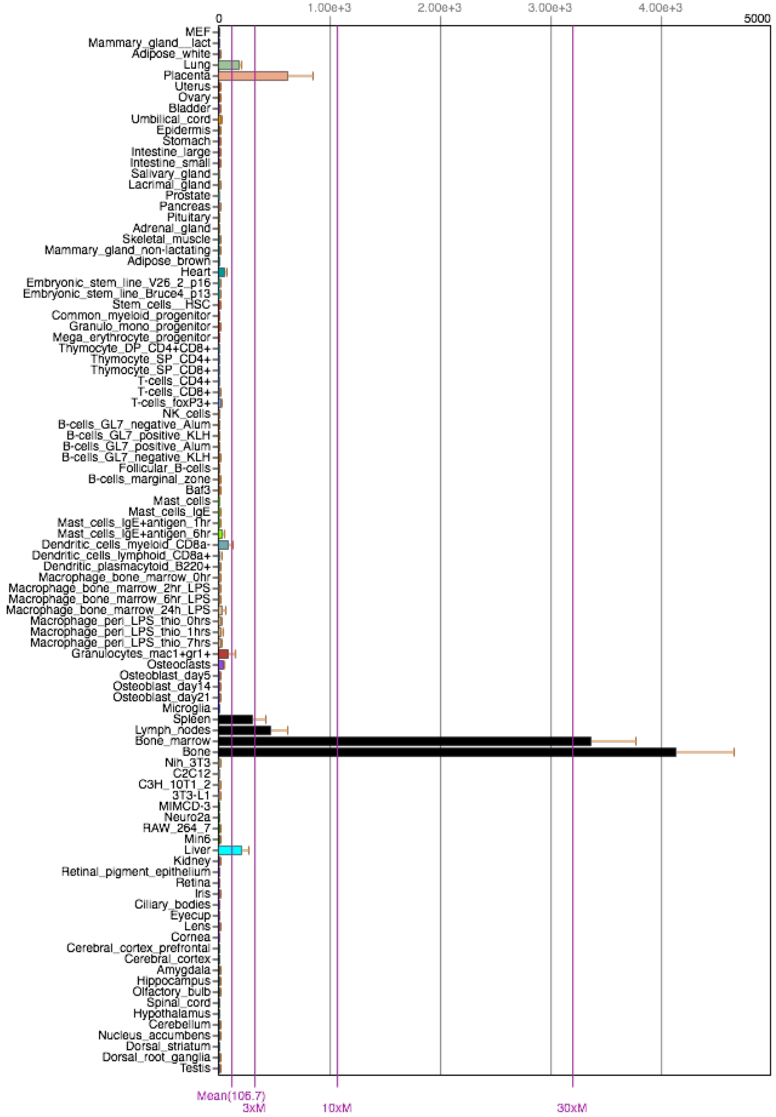

Supporting Information Figure 7:

Kate L. Lowe et al. (2015)

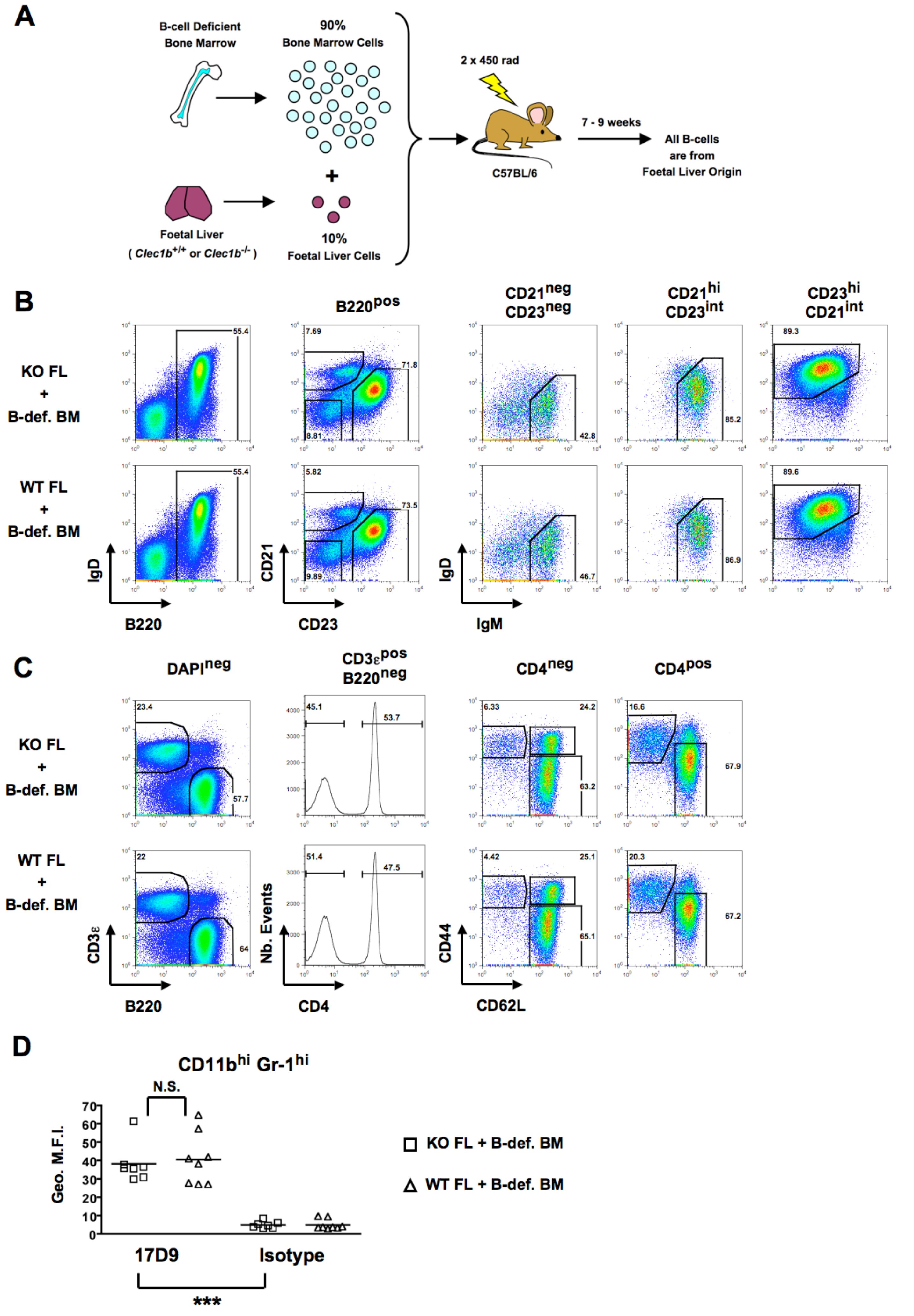

Table 1: List of antibodies used for the flow cytometry analysis

Mouse antigen recognised and fluorophore	Clone	Provider
CD19-APC	1D3	eBiosciences
B220-PECF594	RA3-6B2	BD Biosciences
CD3ε-PEcy7	145-2C11	eBiosciences
CD11c-PE	N148	eBiosciences
CD8α-PE	53-6.7	BD Biosciences
F4/80-PE	BM8	eBiosciences
CD41-PE	MWReg30	eBiosciences
CD8α-APC	53-6.7	eBiosciences
CD4-A647	RM4-5	BD Biosciences
CD19-PE	1D3	eBiosciences
NKp46-PEcy7	29A1.4	Biolegend
NK1.1-PEcy7	PK136	eBiosciences
CD3ε-APC	145-2C11	eBiosciences
F4/80-APC	BM8	eBiosciences
CD11b-FITC	M1/70	eBiosciences
CD11c-PEcy7	N148	eBiosciences
Dead Cells - DAPI	-	AbD Serotec
CD45-APC780	30-F11	eBiosciences
B220-PETxR	RA3-6B2	BD Biosciences
CD11b-PerCPcy5.5	M1/70	eBiosciences
Gr-1-APC	RB6-8C5	eBiosciences
CD19-APCcy7	1D3	BD Biosciences
CD45.2-e780	104	eBiosciences
IgD-A647	11-26	eBiosciences
B220-eFluor450	RA3-6B2	eBiosciences
IgM-PEcy7	11/41	eBiosciences
CD23-PE	B3B4	BD Pharmingen
CD21/35-FITC	7G6	eBiosciences
CD4-FITC	GK1.5	eBiosciences
CD44-PE	1M7	eBiosciences
CD62L-PE	MEL-14	eBiosciences
CLEC-2-FITC	17D9	AbD Serotec
CLEC-2 (purified)	17D9	Caetano Reis e Sousa (Cancer Research UK, London)
CLEC-2 (purified)	INU1	Bernhard Nieswandt (University of Würzburg, Germany)
Isotype rat IgG2b-FITC	MCA1125FT	AbD Serotec
Isotype rat IgG2b (purified)	-	R&D Systems
Isotype rat IgG1k	400402	Biolegend
