# Supplementary material for: The expression of mouse CLEC‐2 on leucocyte subsets varies according to their anatomical location and inflammatory state
Source: Eur J Immunol. 2015 Aug 12;45(9):2484–93. doi: 10.1002/eji.201445314 (PMC4737233; doi:10.1002/eji.201445314)
Supplement: Supplementary file 2 — Peer review correspondence [file EJI-45-2484-s002.pdf]

Peer review correspondence

The expression of mouse CLEC-2 on leucocyte subsets varies according to their anatomical location and inflammatory state

Kate L. Lowe, Leyre Navarro-Nuñez, Cécile Bénézech, Saba Nayar, Bethany L. Kingston, Bernhard Nieswandt, Francesca Barone, Steve P. Watson, Christopher D. Buckley and Guillaume E. Desanti

Corresponding author: Guillaume E. Desanti, Centre for Translational Inflammation Research, Rheumatology Research Group, University of Birmingham, Birmingham, United Kingdom

---

Review Timeline:	Submission date:	4 November 2014
	First editorial decision:	12 December 2014
	First revision received:	22 April 2015
	Second editorial decision:	21 May 2015
	Second revision received:	6 June 2015
	Accepted:	10 July 2015

Handling Executive Committee members: Prof. Silvano Sozzani

Please note that the correspondence below does not include the standard editorial instructions regarding preparation and submission of revised manuscripts, only the scientific revisions requested and addressed.

First Editorial Decision – 12 December 2014

Dear Dr. Desanti,

Manuscript ID eji.201445314 entitled "The expression of mouse CLEC-2 on leucocyte subsets varies according to their anatomical location and inflammatory state" which you submitted to the European Journal of Immunology has been reviewed. The comments of the referees are included at the bottom of this letter.

A revised version of your manuscript that takes into account the comments of the referees will be reconsidered for publication.

European Journal of Immunology

You should also pay close attention to the editorial comments included below. *In particular, please edit your figure legends to follow Journal standards as outlined in the editorial comments. Failure to do this will result in delays in the re-review process.*

Please note that submitting a revision of your manuscript does not guarantee eventual acceptance, and that your revision will be re-reviewed by the referees before a decision is rendered.

If the revision of the paper is expected to take more than three months, please inform the editorial office. Revisions taking longer than six months may be assessed by new referees to ensure the relevance and timeliness of the data.

Once again, thank you for submitting your manuscript to European Journal of Immunology and we look forward to receiving your revision.

Yours sincerely,
Karen Chu

On behalf of Prof. Silvano Sozzani

Dr. Karen Chu
Editorial Office
European Journal of Immunology
e-mail: ejied@wiley.com
www.eji-journal.eu

****

Reviewer: 1

Comments to the Author
In this manuscript, the authors aimed to determine which hematopoietic-derived cell types other than platelets express CLEC-2 in mice. They found that CLEC-2 is expressed on peripheral B cells and CD11bhi Gr-1hi myeloid cells. At resting state, CLEC-2 is not on any type of immune cells in SLOs. However, after LPS challenge, CLEC-2 is expressed on conventional DCs in MLNs. In addition, no changes in mRNA expression of Clec2 in cDC in MLNs was detected with and without LPS challenge.

This is an important study. However, the results require more vigorous tests because it is highly possible that the CLEC-2 expression on some cell types is resulted from association of CLEC-2 expressing

European Journal of Immunology

platelet-derived microparticles. Therefore, flow cytometry analysis of CLEC-2 on various WT cells in the presence of EDTA and/or a P-selectin blocking antibody as well as cells from mice specifically lacking platelet CLEC-2 should be performed.

Reviewer: 2

Comments to the Author

In the manuscript " The expression of mouse CLEC-2 on leucocyte subsets varies according to their anatomical location and inflammatory state" by Lowe et al. the authors investigate the expression of CLEC-2 on mouse leukocytes using the 17D9 antibody. They compare leukocytes from Clec1bfl/fl mice with leukocytes from tamoxifen induced Clec1b-/- mice and leukocytes derived from wild type mice irradiated and reconstituted with Clec1b+/+ and Clec1b-/- embryo foetal liver. The authors show that CLEC-2 is constitutively expressed on platelet, circulating CD11bhighGR1high cells and circulating B cells. They also show that secondary lymphoid organs resident B-lymphocytes and CD11bhighGR1high cells are negative. Moreover, the authors show that CLEC-2 expression can be induced in a subset of DCs in LPS-mediated inflammatory conditions while resting DCs are negative.

The work is well performed and the data presented fully support the conclusions of the authors.

Recent works have proposed very important roles for CLEC-2, including for instance the regulation of lymph node expansion in inflammatory conditions. Therefore, an accurate evaluation of CLEC-2 expression by leukocytes in resting and inflammatory conditions in different anatomical regions, although descriptive, is relevant for the scientific community to better evaluate possible CLEC-2 functions.

Reviewer: 3

Comments to the Author

The manuscript by Desanti describes the generation of a CLEC2 ko mouse in order to reveal the expression of CLEC2 on leucocyte subsets varies according to their anatomical location and inflammatory state. They clearly describe leukocyte subsets to express the CLEC2 receptor, but little functional data or discussions are given nor the consequences of the regulated expression of this receptor for their function.
The novelty lies in the CLEC-2 expression on B cells, but one would like to see what the functional consequences of this expression are. Any addition of function data and binding of the counterstructure of CLEC2 receptor would strenghten the paper. As it stands, the paper is too descriptive.

European Journal of Immunology

First revision – authors' response – 22 April 2015

Reviewer: 1

R.1: 'This is an important study. However, the results require more vigorous tests because it is highly possible that the CLEC-2 expression on some cell types is resulted from association of CLEC-2 expressing platelet-derived microparticles.'
G.E.D.: We are grateful to the Reviewer n°1 for stressing the importance of this study. Further to Reviewer n°1's comments, we acknowledged that some of our results depicting CLEC-2 expression on leucocytes might be artefacts coming from their association with platelet-derived microparticles.
In order to address this point, we performed new in vivo reconstitution experiments to generate animals with a complete Clec1b deficiency restricted to B-lymphocytes. The results of these experiments are now given in the newly generated Figure 3 and Supplemental Information Figure 6. These new results are reported in the Abstract (page 2), the Results and discussion (pages 10-11) and the Concluding remarks (page 12).
As these new results confirm the Reviewer n°1's intuition by demonstrating that peripheral blood B-lymphocytes only present an exogenous CLEC-2 on their surface, we are very thankful to the Reviewer n°1 for his/her comment as it greatly improved the quality of our manuscript.

R.1: '[…] flow cytometry analysis of CLEC-2 on various WT cells in the presence of EDTA […] should be performed '
G.E.D.: The initial version of the manuscript was already mentioning in the Material and methods (page 14, in Antibodies, FACS and cell sorting) that all our flow cytometry staining and analysis were performed in a buffer containing 2m EDTA. Moreover, we mentioned in page 12 that the blood isolated by tail bleed were collected in a 20mM EDTA solution (page 12, in Material and methods).
In the new version of the manuscript, we added in Material and methods (pages 13-14, in Tissues sampling and preparation) that the blood isolated from the inferior vena cava were collection in a 20mM EDTA solution. We apologize for this initial omission.
We believe that the information provided in the revised version of the manuscript clearly state the presence of EDTA during our experimental procedures.

R.1: '[…] flow cytometry analysis of CLEC-2 on various WT cells in the presence of […] P-selectin blocking antibody […] should be performed '
G.E.D.: We took the Reviewer n°1's suggestion into consideration during the preparation of the new version of this manuscript.
We first performed FACS staining including anti-CD41, anti-CD42b and anti-CD62P (i.e., anti-P-selectin) in order to detect the presence of such platelet-derived markers on leucocytes. Unfortunately, this co-

European Journal of Immunology

staining seems to have led to an antibody-mediated platelet-leucocyte aggregation. As a consequence, the results obtained were not suitable for publication.

Taking into account the un-reliability of this FACS staining and the fact that the totality of our FACS staining have been performed in presence of 2mM EDTA (as mentioned in the previous paragraph), we concluded that engineering Clec1b deficient animals was the best approach to tackle this issue (as mentioned in the first paragraph and in the paragraph below).

R.1: ''[…] flow cytometry analysis of CLEC-2 […] from mice specifically lacking platelet CLEC-2 should be performed.'

G.E.D.: In order to address this point, we considered using the PF4-cre x Clec1bfl/fl mice we initially described in our previous publication (Bénézech C., Nayar S., et al. Blood 2014. 123: 3200-3207). However, two independent studies have now reported that the PF4-cre is not restricted to the megakaryocyte/platelet lineage (please see: Calaminus, S. D., Guitart, A. V., et al. Lineage tracing of Pf4-Cre marks hematopoietic stem cells and their progeny. PLoS One 2012. 7: e51361. and Pertuy, F., Aguilar, A., et al. Broader expression of the mouse platelet factor 4-cre transgene beyond the megakaryocyte lineage. J Thromb Haemost 2015. 13: 115-125.). These works demonstrate that the PF4-cre transgene could also affect the myeloid and B-lymphocyte lineages.

In order to bypass this problem and in an attempt to address the Reviewer n°1's request, we generated animals with Clec1b full deficiency restricted to B-lymphocytes. We believe that this strategy provides answer to some of the Reviewer n°1's questions. It allows us to demonstrate that CLEC-2 on peripheral blood B-lymphocytes is stemming from other cell types as suggested by Reviewer n°1. The results of these experiments are now given in the newly generated Figure 3 and Supplemental Information Figure 6. These new results are reported in the Abstract (page 2), the Results and discussion (pages 10-11) and the Concluding remarks (page 12).

Reviewer: 2

R.2: 'The work is well performed and the data presented fully support the conclusions of the authors. […] Therefore, an accurate evaluation of CLEC-2 expression by leukocytes in resting and inflammatory conditions in different anatomical regions, although descriptive, is relevant for the scientific community to better evaluate possible CLEC-2 functions.'

G.E.D.: We are grateful to the Reviewer n°2 for his/her positive appreciation about our work and its relevance to the scientific community.

In an attempt to associate our observations with an immunological function, we have now addressed the effect of a B-lymphocyte-restricted Clec1b deficiency on blood-lymph flow segregation, the maintenance of HEVs in secondary lymphoid organs and lymphocyte homeostasis. The results of these experiments are now given in the newly generated Figure 3 and Supplemental Information Figure 6. These new results are

reported in the Abstract (page 2), the Results and discussion (pages 10-11) and the Concluding remarks (page 12).

Reviewer: 3

R.3: 'They clearly describe leukocyte subsets to express the CLEC2 receptor, but little functional data […]. The novelty lies in the CLEC-2 expression on B cells, but one would like to see what the functional consequences of this expression are. Any addition of function data and binding of the counterstructure of CLEC2 receptor would strenghten the paper. As it stands, the paper is too descriptive.'
G.E.D.: We appreciate Reviewer n°3's opinion on the absence of functional data in the previous version of our manuscript. We agree with Reviewer n°3 that the main novelty of our work lies in the expression of CLEC-2 by peripheral blood B cells and that it is important to understand the functional consequences coming with this expression.
In an attempt to understand the functional significance of CLEC-2 on B-lymphocytes, we generated animals with a complete Clec1b deficiency restricted to B-lymphocytes. By comparing these animals to relevant controls, we analyzed the role of B-lymphocytes' Clec1b expression on blood-lymph flow segregation, the maintenance of HEVs in secondary lymphoid organs and lymphocyte homeostasis. It appeared that Clec1b expression by B-lymphocytes play almost no role (with the exception of CD4+ T-lymphocytes recruitment in the spleen) in these physiological mechanisms. We managed to explain this absence of function by the fact that: 1) B-lymphocytes are not the main source of CLEC-2 in the blood but platelets are; 2) CLEC-2 found on the surface of peripheral blood B-lymphocytes is not produced by B-lymphocytes themselves but stem from other cell types. The transfer of CLEC-2 onto B-lymphocytes is probably mediated by cell-derived microparticles.
We hope that these new results will meet Reviewer n°3's requirements. The results of these experiments are now given in the newly generated Figure 3 and Supplemental Information Figure 6. These new results are reported in the Abstract (page 2), the Results and discussion (pages 10-11) and the Concluding remarks (page 12).

R.3: 'They clearly describe leukocyte subsets to express the CLEC2 receptor, but little […] discussions are given nor the consequences of the regulated expression of this receptor for their function.'
G.E.D.: We were surprised to read that Reviewer n°3 considers that we gave little discussion about the consequences of the regulated expression of CLEC-2 by leucocytes. The initial version of our manuscript discussed in pages 8 and 9 the possible physiological requirements driving CLEC-2 down-regulation by B-lymphocytes and CD11bhigh Gr-1high cells when they enter into secondary lymphoid organs (in sub-sections: Most SLO-resident leucocytes do not express CLEC-2/Clec1b at steady-state and Most SLO-resident leucocytes remain CLEC-2-negative following LPS stimulation). This point was further discussed in the Concluding remarks (in page 11). We also discussed in pages 9 and 10 that the CLEC-2 up-

regulation on dendritic cells was depending on the anatomical environment and the nature of immunogen (in sub-section: MLN but not splenic CD11bhigh cDCs acquire CLEC-2 following LPS stimulation).
The new version of the manuscript maintains the essence of these previously discussed points. We have also added new thoughts about the presence of CLEC-2 on peripheral blood B-lymphocytes and its potential role on blood-lymph flow segregation, the maintenance of HEVs in secondary lymphoid organs and lymphocyte homeostasis from the new results we generated for this re-submission. These new thoughts are reported in the Results and discussion (pages 10-11) and the Concluding remarks (page 12).

Second Editorial Decision – 21 May 2015

Dear Dr. Desanti,

Thank you for submitting your revised manuscript ID eji.201445314.R1 entitled "The expression of mouse CLEC-2 on leucocyte subsets varies according to their anatomical location and inflammatory state" to the European Journal of Immunology. Your manuscript has been re-reviewed and the comments of the referees are included at the bottom of this letter.

Although referee 1 has now recommended publication, some revisions to your manuscript have been requested. Therefore, I invite you to respond to the comments of referee 1 and revise your manuscript accordingly.

You should also pay close attention to the editorial comments included below. *In particular, please edit your figure legends to follow Journal standards as outlined in the editorial comments. Failure to do this will result in delays in the re-review process.*

If the revision of the paper is expected to take more than three months, please inform the editorial office. Revisions taking longer than six months may be assessed by new referees to ensure the relevance and timeliness of the data.

Once again, thank you for submitting your manuscript to European Journal of Immunology. We look forward to receiving your revision.

Yours sincerely,
Karen Chu

on behalf of Prof. Silvano Sozzani

Peer review correspondence

Dr. Karen Chu
Editorial Office
European Journal of Immunology
e-mail: ejied@wiley.com
www.eji-journal.eu

****

Reviewer: 1

Comments to the Author
The authors have made a very good effort to address reviewers' comments. As a result, the revised manuscript is stronger. But a few issues remain to be addressed
1. B cell specific deletion of CLEC-2 supports that B cells do not express endogeneous CLEC-2, but does not tell the origin of CLEC-2. Therefore, the conclusion that peripheral B cells present microparticles-derived CLEC-2 is an over-interpretation.
2. The authors are encouraged to address whether the CLEC-2 on CD11bhighGr1high cells is derived from exogenous sources.
3. In general, the manuscript needs a significant revision for its clarity. For example, while the 2nd paragraph of page 13 states that 17D9 was used for investigating CLEC-2 on leukocytes, the 1st sentence of page 14 describes 17D9 binding to CD11bhighGr1high in a CLEC-2-independent manner. In addition, quite a few figures are mis-matched with their result descriptions; for example, results referred to as Supplemental Figure 6C should be Supplemental Figure 7D, and data referred to as Figure 6E might actually be Figure 6A or B.

Second revision – authors' response – 6 June 2015

Referee: 1

R.1: 'The authors have made a very good effort to address reviewers' comments. As a result, the revised manuscript is stronger[…] .'
G.E.D.: We are grateful to Referee n°1 for stressing the effort we made to meet the referees' requests and the impact of these efforts on the quality of the manuscript.

European Journal of Immunology

R.1: '1. B cell specific deletion of CLEC-2 supports that B cells do not express endogeneous CLEC-2, but does not tell the origin of CLEC-2. Therefore, the conclusion that peripheral B cells present microparticles-derived CLEC-2 is an over-interpretation. '

G.E.D.: We thank the Referee n°1 for pointing out this over-interpretation and we have modified the manuscript accordingly. We have now mentioned in the manuscript that:

i) '[…] we demonstrate for the first time that peripheral blood B lymphocytes present exogenously derived CLEC-2 and […]' (Abstract page 3).

ii) '[…] we propose that the CLEC-2 molecules detected on the surface of circulating B lymphocytes are derived from MHC-II antigen presentation, trogocytosis or exosomes/microparticles attached to the B cell membrane {Ahmed, 2011 #69}.' (Results and Discussion, page 12).

iii) 'These B cells do not produce CLEC-2 but likely acquire CLEC-2 molecules from other yet uncharacterised cell types.' (Concluding remarks, page 13).

R.1: '2. The authors are encouraged to address whether the CLEC-2 on CD11bhigh Gr1high cells is derived from exogenous sources.'

G.E.D.: We considered investigating this question while we were preparing our manuscript for re-submission between December 2014 and April 2015. However, the expression of CLEC-2 on these cells has been reported previously by Kerrigan et al. (2009) and Mourao-Sa et al. (2011), as well as the reports of CLEC-2 expression on monocytes by Tang et al. (2010) while Fossum et al. reported a medium level of Clec1b transcript expression in rat granulocytes. It would have been possible for us to check for Clec1b transcript levels on CD11bhigh Gr1high cells by quantitative PCR but we did not have the possibility to generate myeloid-restricted Clec1b-deficient animals to fully investigate this question at the protein and functional levels. In this manuscript we report for the first time that B lymphocytes have CLEC-2 on their surface. For this reason we focussed our study to fully investigate this novel finding and decided to leave aside for the moment the circulating CD11bhigh Gr1high population.

R.1: '3. In general, the manuscript needs a significant revision for its clarity. For example, while the 2nd paragraph of page 13 states that 17D9 was used for investigating CLEC-2 on leukocytes, the 1st sentence of page 14 describes 17D9 binding to CD11bhighGr1high in a CLEC-2-independent manner.'

G.E.D.: We apologise for the confusion. On page 13 we describe the weakness of using INU1 to detect CLEC-2 and further demonstrate the specificity of 17D9 on platelets and leucocytes under resting conditions. On pages 14 and 16 we describe an interesting phenomenon whereby 17D9 binds to some leucocytes independently of CLEC-2 following LPS challenge. We feel this is an extremely important point to make based on the conclusions from previously published literature that did not take this into account due to the lack of CLEC-2-negative control cells (as mentioned once in the second paragraph of page 16 to avoid duplication). We hope we have clarified this query and justified its inclusion in the manuscript.

European Journal of Immunology

R.1: '3. […] quite a few figures are mis-matched with their result descriptions; for example, results referred to as Supplemental Figure 6C should be Supplemental Figure 7D, and data referred to as Figure 6E might actually be Figure 6A or B.'

G.E.D.: We indeed made a few mistakes while editing the final version of our re-submitted manuscript. We apologise for this and are grateful to the Referee for raising our attention to them. These mistakes have now been corrected.

Third Editorial Decision – 17 June 2015

Dear Dr. Desanti,

It is a pleasure to provisionally accept your manuscript entitled "The expression of mouse CLEC-2 on leucocyte subsets varies according to their anatomical location and inflammatory state" for publication in the European Journal of Immunology. For final acceptance, please follow the instructions below and return the requested items as soon as possible as we cannot process your manuscript further until all items listed below are dealt with.

Please note that EJI articles are now published online a few days after final acceptance (see Accepted Articles: http://onlinelibrary.wiley.com/journal/10.1002/(ISSN)1521-4141/accepted). The files used for the Accepted Articles are the final files and information supplied by you in Manuscript Central. You should therefore check that all the information (including author names) is correct as changes will NOT be permitted until the proofs stage.

We look forward to hearing from you and thank you for submitting your manuscript to the European Journal of Immunology.

Yours sincerely,
Karen Chu

on behalf of Prof. Silvano Sozzani

Dr. Karen Chu
Editorial Office
European Journal of Immunology
e-mail: ejied@wiley.com
www.eji-journal.eu
